# Flash Communication: Strained and Bimetallic Structures of Rhodium and Iridium Germyl Complexes with Phosphinoamido Ligands

**DOI:** 10.1021/acs.organomet.5c00403

**Published:** 2025-11-25

**Authors:** Sonia Bajo, Marta Fernández-Buenestado, Joaquín López-Serrano, Jesús Campos

**Affiliations:** Instituto de Investigaciones Químicas (IIQ), Departamento de Química Inorgánica and Centro de Innovación en Química Avanzada (ORFEO-CINQA), Consejo Superior de Investigaciones Científicas (CSIC) and Universidad de Sevilla, Avenida Américo Vespucio 49, 41092 Sevilla, Spain

## Abstract

In the coordination chemistry of heavier tetrylenes (:ER_2_, where E = Si, Ge, Sn, Pb), chelating P,N-donor ligands occupy a privileged position, though phosphinoamido ligands, [R_2_P–NR′]^−^, have been barely investigated. Herein, we report the synthesis and structural characterization of such complexes, whose structures drastically differ depending on the group 9 metal precursor used. Strained four-membered {P–N–Ge–M} metallacycles (M = Rh, Ir) are produced from the reaction of phosphinoamido germylenes with [MCl_2_Cp*]_2_ precursors (Cp* = η^5^-C_5_Me_5_). At variance, [MCl­(COD)]_2_ dimers are not broken apart; instead, they afford bimetallic species featuring bridging phosphinoamido–germyl and chloride ligands between the two metals. All new compounds were isolated on a preparative scale and spectroscopically characterized and their structures confirmed by X-ray diffraction. Computational studies support the σ-donor character of the Ge–M interaction and the absence of significant π-backbonding.

## Graphical abstract

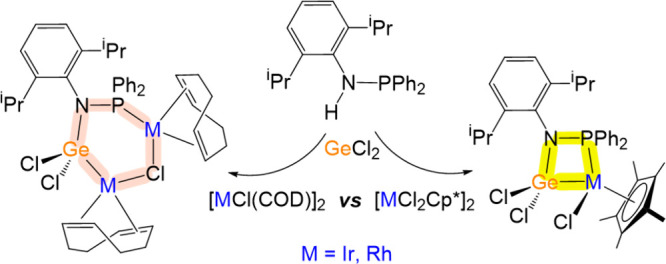

The chemistry of heavier tetrylenes (:ER_2_, E = Si, Ge, Sn, Pb) is highly distinct from that of traditional carbenes. They tend to exhibit transition metal-like behavior, particularly in their ability to activate small molecules.^1^ Besides, when bonded to transition metals, they offer opportunities for synergistic interactions that may result in enhancing the reactivity of the resulting complexes,^2^ allowing for the cooperative activation and functionalization of numerous molecules,^3^ with direct implications in catalysis. Synthetically, the use of chelating ligands containing both a nitrogen and a phosphorus donor (i.e., P,N ligands) has been quite popular.^4^ Based on hard–soft acid–base (HSAB) principles,^5^ the hard N-donor coordinates to the tetrylene, while the soft P-donor binds the transition metal. In turn, the overall ligand geometry is instrumental for complex stability and to allow tuning of the proximity between the two sites, thereby the possibility of forming a direct tetrylene-transition metal bond.

Most often, the N- and P-donors are separated by one or two atoms, leading to 5- or 6-membered metallacycles, respectively^4^ (Figure 1a). We recently investigated the effect of elongating the spacer toward a 7-membered metallacycle that ensured stabilizing an uncommon germylene form^6^ (Figure 1a). At variance, eliminating the spacer between N- and P-donors in phosphinoamido ligands ([R_2_P–NR′]^−^) would result in more strained 4-membered metallacycles of potentially enhanced reactivity. This has already been successfully exploited by Thomas in bimetallic complexes containing two transition metals.^7^ In the case of germanium, its sole coordination to phosphinoamido ligands has been investigated only to some extent (Figure 1b).^8^ Nonetheless, to the best of our knowledge, there is only one example, reported by Xi, which features a strained four-membered {GeNPNi} ring,^9^ in which the germylene is further stabilized by an amidinate ligand (Figure 1a).

**Figure 1:**
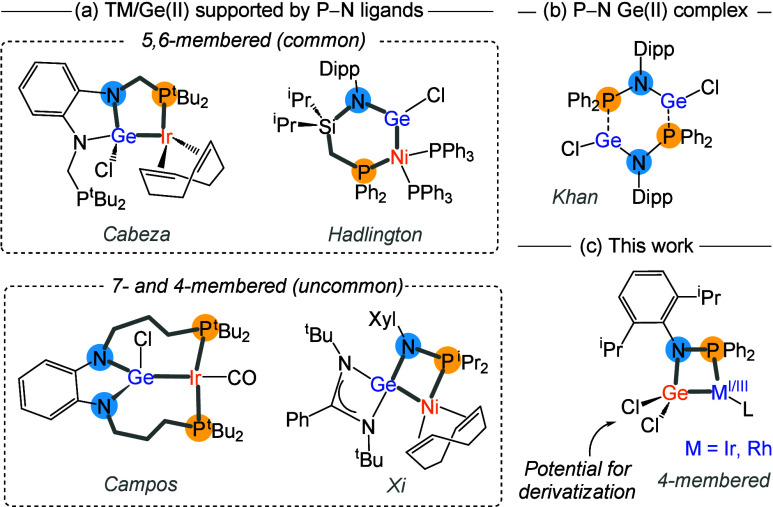
(a) Representative examples of previously reported transition metal/germanium­(II) complexes bearing chelating P,N ligands leading to 5- and 6-membered metallacycles, and the only two examples of more exotic 7- and 4-member metallacycles. (b) Well-defined germylene stabilized by a P–N ligand. (c) This work: uncommon 4-membered M/Ge­(II) metallacycles without additional ligands on germanium (Dipp = C_3_H_6_-2,6-^*i*^Pr_2_; Xyl = C_6_H_3_-3,5-Me_2_).

On these grounds, we wondered whether it would be possible to access similarly strained species but without additional ligands on the germanium center to allow for later and more versatile functionalization (Figure 1c). Herein, we describe our results on the synthesis and characterization of such kind of strained species containing germanium coordinated to iridium and rhodium fragments.

We chose the phosphinoamido germylene dimer represented in Figure 1b and described by Khan and co-workers^8b^ as an ideal precursor to access our targeted 4-membered metallacycles. As previously described, treatment of phosphinoamine 2,6-^i^Pr_2_C_6_H_3_(H)­N–PPh_2_ with ^n^BuLi overnight ensures complete deprotonation of the NH group. Subsequently, one equivalent of GeCl_2_·dioxane suspended in toluene was added at −78 °C, resulting in the formation of the previously reported germylene dimer, confirmed by a ^31^P­{^1^H} NMR resonance at 48.3 ppm.^8b^ Without further isolation or purification, the reaction mixture was treated with the corresponding transition metal precursor, in particular, [MCl_2_(Cp*)]_2_ (Cp* = η^5^-C_5_Me_5_; M = Rh, Ir) or [MCl­(COD)]_2_ (COD = 1,5-cyclooctadiene; M = Rh, Ir), to compare the structural and bonding effects between rhodium and iridium and between oxidation states I and III. All new complexes were isolated in moderate yields (50–60%) at a half-gram scale.

First, the reaction with the precursors [MCl_2_(Cp*)]_2_ led to the formation of new complexes **1a** (Rh) and **1b** (Ir) (Scheme 1). This was confirmed by the appearance of new ^31^P­{^1^H} NMR resonances, a doublet at 81.8 ppm for **1a** with a ^1^ *J*_PRh_ = 139 Hz, and a singlet at 43.9 ppm for **1b** (cf. 48.3 for the germylene precursor). The formation of complexes **1a** and **1b** restricts the amide ring’s rotational freedom which is reflected by four ^1^H NMR resonances for the CH_3_ groups of the isopropyl moieties (**1a**: 1.45, 1.41, 0.54, and 0.27 ppm; **1b**: 1.45, 1.42, 0.52, and 0.28 ppm; cf. 1.14 ppm for the free ligand). In contrast, a single ^1^H resonance at 1.30 ppm is recorded for the Cp* ligand in compounds **1a** and **1b**, slightly high-shifted compared to their precursors (1.26 ppm).

**Scheme 1:**
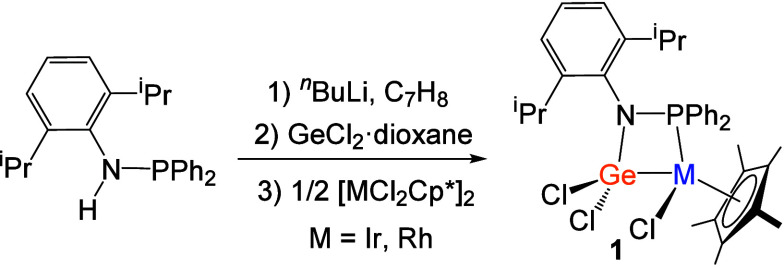
Synthesis of Complexes [(PN)­Cl_2_Ge–MClCp*] (M = Rh (**1a**) or Ir (**1b**); PN = DippNPPh_2_)

The structures of compounds **1a** and **1b** were confirmed by X-ray diffraction analysis (Figure 2, **1a**; Figure S14, **1b**). Both structures feature similar metrics of the {P–N–Ge–M} core. The Rh–Ge and Ir–Ge distances of 2.3656(5) and 2.3799(7) Å are slightly shorter than the sum of their covalent radii (2.62 and 2.61 Å, respectively),^10^ but within the expected range for single M–Ge bonds. Comparative analysis with related rhodium^11^ and iridium^4b^ germylene compounds stabilized by other type of P,N-containing ligands reveal similar M–Ge, N–Ge and M–P bond distances. However, compounds **1** exhibit a more constrained 4-membered core structure due to the direct bond between nitrogen and phosphorus. This is evinced by the P–M–Ge bond angles of 69.33(3)° (**1a**) and 69.48(5)° (**1b**) compared to the wider values (80–87°) reported for the related examples based on extended rings,^4b,11^ and even smaller than the 4-membered nickel species reported by Xi (74–74°),^9^ in line with the smaller size of the nickel center.

**Figure 2:**
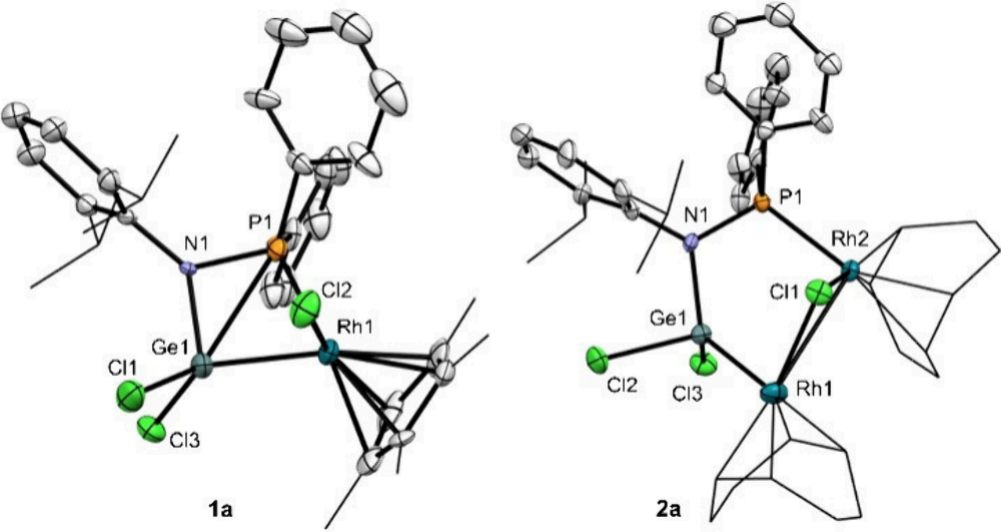
ORTEP diagrams for complexes **1a** and **2a**. Hydrogen atoms have been excluded, and isopropyl groups and methyl termini of the Cp* and COD ligands are represented in wireframe format for clarity. Thermal ellipsoids are set at 50% probability.

We also attempted the corresponding reaction with the [MCl­(COD)]_2_ dimers. At variance with the above results, the stoichiometric reactions between the phosphinoamine ligand and 1/2 equiv of the corresponding dimer did not led to completion under similar conditions. Instead, approximately half of Khan’s germylene precursor remained unaltered upon consumption of the metallic dimers. This observation suggested that one molecule of the dimer was coordinated to the germylene without dissociating into monomers, as occurred for the formation of compounds **1**. Indeed, the major products of these reactions, compounds **2a** and **2b**, were better synthesized by using 1 equiv of the dimer per germylene unit. The structures of these bimetallic species could be corroborated by X-ray diffraction analysis (*vide infra*). They feature two transition metal atoms bridged by one μ-chlorido and one phosphinoamido-germyl ligand (Scheme 2). The formation of these new complexes was monitored by the appearance of new ^31^P­{^1^H} NMR resonances, a doublet at 63.7 ppm (^1^ *J*_PRh_ = 152.4 Hz) for **2a** and a singlet at 48.9 ppm for **2b**. A very similar ^1^H NMR pattern, with eight distinctive olefinic resonances in the 4–7 ppm region due to the presence of two inequivalent coordinated COD ligands (see Figure S13), was diagnostic of identical structures between **2a** and **2b**.

**Scheme 2:**
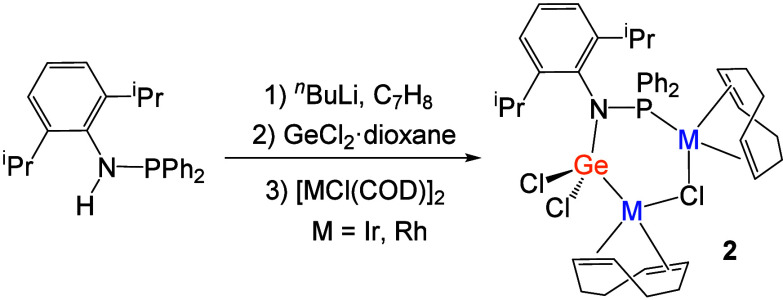
Synthesis of Complexes [(PNGe)­(M­(COD))_2_(μ-Cl)] (M = Rh (**2a**) or Ir (**2b**); PN = DippNPPh_2_)

The structure of compound **2a** was confirmed by an X-ray diffraction analysis (Figure 2). According to the X-ray data, the two rhodium centers are in a distorted square planar environment defined by the COD ligand, the μ-Cl and either the germyl or phosphine fragments, in both cases in agreement with a Rh­(I) formulation. The Rh···Rh distance is 3.299(1) Å, substantially deviated from the sum of their covalent radii (2.84 Å),^10^ and consistent with the absence of Rh–Rh bonding, in line with our computational analysis (*vide infra*). The Rh1–Ge1 and Rh2–P1 bond distances of 2.407(1) and 2.314(3) Å, respectively, are consistent with expected values.^11^ The Ge atom is in a distorted tetrahedral environment with angles of 125.7(2)° for N–Ge–Rh1, 97.3(1)° for Cl3–Ge–Cl2 and 111.41(9)° for Rh1–Ge–Cl2, as expected for a germyl formulation and notably more distorted than in the more strained compound **1a**.

We carried out computational analysis to shed some light on the bonding between the phosphinoamido chlorogermyl moiety and the transition metals. Geometry optimizations at the DFT-B3PW91-D3BJ/def2-TZVP+SDD level of theory (see the Supporting Information for further details) reproduced well the solid-state molecular geometries of complexes **1a**, **1b** and **2a**, obtained by XRD analysis. For instance, in **1a** the computed Rh–Ge and Rh–P bond distances present values of 2.38 and 2.28 Å (*c.f.* 2.3656(5) and 2.315(1) Å, respectively, from the X-ray structure), while the calculated P–Rh–Ge angle of 69.63° is almost equal to the experimental one (69.33(3)°). Natural Bond Orbital (NBO) analysis of compounds **1** revealed similar Wiberg Bond Indices (WBIs) for the Rh–Ge and Ir–Ge bonds of 0.47 and 0.53, respectively, similar to Rh–P and Ir–P bonds (0.53 and 0.60) in those compounds. The same is observed in species **2** (Rh–Ge, 0.52; Ir–Ge, 0.57; Rh–P, 0.55; Ir–P, 0.59), suggesting in all cases the expected σ-bonding between the P- or Ge-donor and the metal. Indeed, the Rh–Ge bond in **1a** is described in terms of a largely covalent σ interaction between a sd orbital of rhodium (40%) and a sp^0.39^ orbital of germanium (60%), while we could not locate any significant π-backdonation component. These results, in conjunction with analysis of the corresponding natural Localized Molecular Orbitals (NLMO) for species **1** and **2**, are in line with the dative nature of Ge–M bonds, as seen in Figure 3 for **1a** and **2a** (see the Supporting Information for more details). Finally, a WBI of 0.044 between the two Rh centers in **2a** is consistent with the absence of bimetallic bonding. Besides, frontier orbitals of species **1** and **2** are described in the SI (Figures S16 and S17).

**Figure 3:**
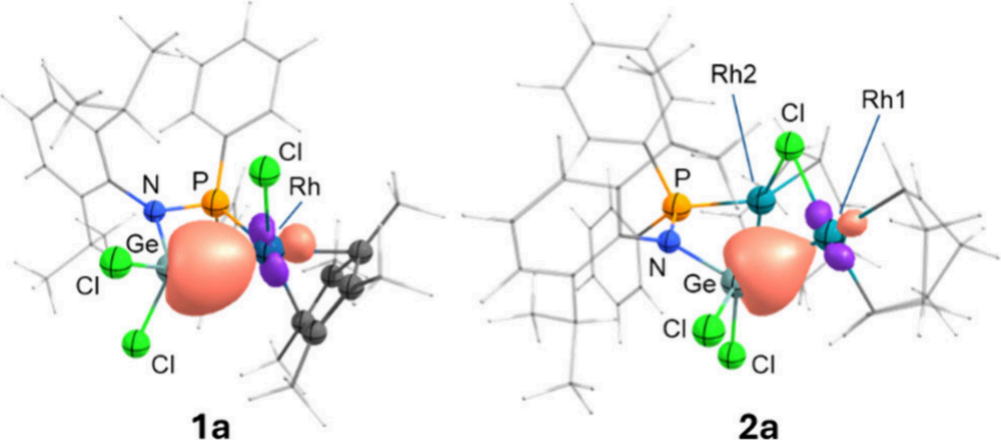
NLMOs for the Rh–Ge σ bond in complexes **1a** and **2a**.

In summary, we have synthesized and characterized a series of rhodium and iridium complexes constructed around phosphinoamido-germylene platforms. While the use of [MCl_2_Cp*] dimers led to strained four-membered {P–N–Ge–M} metallacycles (M = Rh, Ir) characterized by narrow P–M–Ge angles, the addition of [MCl­(COD)]_2_ precursors yield uncommon bimetallic complexes featuring bridging phosphinoamido–germyl and chloride ligands, further highlighting the versatility of this approach. Structural and computational analyses confirm the dative nature of the Ge–M interaction and the absence of π-backbonding. The new complexes were prepared in half-gram scale in good yields, which will facilitate further derivatization at the germyl position via metathesis processes at the Ge–Cl units, thereby providing new platforms for further investigations.

## Supplementary Material





## Notes

Synthesis and characterization of new compounds, NMR spectra, X-ray diffraction studies, and computational studies (PDF)
Computational coordinates (XYZ)
