# Supplementary material for: Flash Communication: Strained and Bimetallic Structures of Rhodium and Iridium Germyl Complexes with Phosphinoamido Ligands
Source: Organometallics. 2025 Nov 25;44(23):2729–32. doi: 10.1021/acs.organomet.5c00403 (PMC12690572; doi:10.1021/acs.organomet.5c00403)
Supplement: Supplementary file 1 [file om5c00403_si_001.pdf]

Supporting Information

Flash Communication: Strained and Bimetallic Structures of Rhodium and Iridium Germyl Complexes with Phosphinoamido Ligands

Sonia Bajo†*, Marta Fernández-Buenestado†, Joaquin López-Serrano, Jesús Campos*

Instituto de Investigaciones Químicas (IIQ), Departamento de Química Inorgánica and Centro de Innovación en Química Avanzada (ORFEO-CINQA), Consejo Superior de Investigaciones Científicas (CSIC) and Universidad de Sevilla. Avenida Américo Vespucio 49, 41092 Sevilla, Spain

sbajo@us.es; jesus.campos@iiq.csic.es

Table of Contents

1. General considerations S1

2. Synthesis and characterization of new compounds S2

3. NMR spectra of new compounds S7

4. Crystal structure determinations S14

5. Computational studies S16

6. References S22

1. General considerations

All preparations and manipulations were performed by using standard Schlenk and glovebox techniques, under an atmosphere of argon and of high purity nitrogen, respectively. All solvents were dried, stored over 4 Å molecular sieves, and degassed prior to use. Toluene (C_7H_8), THF (C_4H_8O), diethyl ether ($(CH_3CH_2)_2O$) and *n*-pentane (C_5H_{12}) were distilled under nitrogen over sodium. C_6D_6 was dried over CaH_2 and distilled under argon. $^{Dipp}ArNHPPh_2$[1] ligand and $[RhCl_2Cp^*]_2$[2], $[IrCl_2Cp^*]_2$[2], $[RhCl(COD)]_2$[3] and $[IrCl(COD)_2]_2$[3] precursors were prepared as described previously. All other reagents were used as received from commercial suppliers. Solution NMR spectra were recorded with Bruker AVANCE NEO-300, AVANCE NEO-400, AVANCE III-400 and AVANCE NEO-500 spectrometers. Spectra were referenced to external $SiMe_4$ (δ: 0 ppm) by using the residual proton solvent peaks as internal standards (^1H NMR experiments), or the characteristic resonances of the solvent nuclei (^{13}C NMR experiments), while ^{31}P was referenced to H_3PO_4. The following abbreviations and their combinations are used: br, broad; s, singlet; d, doublet; t, triplet; m, multiplet. The ^1H and ^{13}C signals were assigned by means of 2D HSQC and HMBC experiments. For elemental analyses a LECO TruSpec CHN elementary analyser was utilized.

2. Synthesis and characterization of new compounds

(κ^N:κ^P-$Ph_2PNDipp$)Cl_2Ge–RhClCp* (Compound 1a)

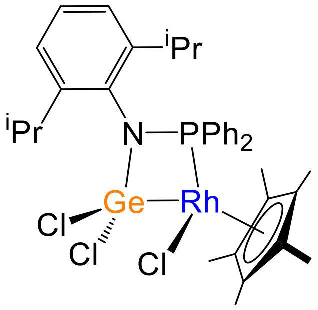

In a J. Young ampoule, DippNHPPh$_2$ (409 mg, 1.13 mmol) was dissolved in toluene (12 mL), reacted with ^{n}BuLi (0.45 mL, 1.13 mmol) at -78 ºC and stirred overnight. A suspension of $GeCl_2$·dioxane (394 mg, 1.7 mmol) in 10 mL of toluene was added dropwise and stirred about 2 hours. A solution of precursor [RhCl$_2$Cp*]$_2$ (350 mg, 0.57 mmol) in 10 mL of toluene was added to the previous mixture and stirred overnight. The resulting mixture was filtered and solvent was removed. The solid obtained was washed with Et_2O (3 x 10 mL) and dried under vacuum to afford an orange-red solid. Crystals suitable for XRD were obtained by slow diffusion of pentane into the compound solution. Yield: 543 mg (63 %).

Anal. Calcd. for $C_{34}H_{42}GeRhCl_3NP_2$: C, 52.5; H, 5.4; N, 1.8. **Found**: C, 53.2; H, 5.9; N, 1.7.

^{1}H NMR (400 MHz, C_6D_6, 298 K): δ = 8.29 (br s, 1H, CH-PPh$_2$), 7.58 (br s, 2H, CH-PPh$_2$), 7.28 (br s, 1H, , CH-PPh$_2$), 7.00-7.13 (m, 3H, CH-Ar^{Dipp}), 6.92 (br s, 4H, , CH-PPh$_2$), 6.68 (br s, 1H, , CH-PPh$_2$), 6.45 (br s, 1H, , CH-PPh$_2$), 4.36 (m, 1H, CH-isopropyl), 4.29 (m, 1H, CH-isopropyl), 1.45 (d, $^3J_{HH}$ = 6.7 Hz, 3H, CH$_3$-isopropyl), 1.41 (d, $^3J_{HH}$ = 6.6 Hz, 3H, CH$_3$-isopropyl), 1.30 (d, $^4J_{HP}$ = 3.1 Hz, 15H, Cp*), 0.54 (d, $^3J_{HH}$ = 6.8 Hz, 3H, CH$_3$-isopropyl), 0.27 (d, $^3J_{HH}$ = 6.5 Hz, 3H, CH$_3$-isopropyl).

^{31}P{^{1}H} NMR (160 MHz, C_6D_6, 298K): δ = 81.8 (d, $^1J_{PRh}$ = 139 Hz, Rh-PPh$_2$).

^{13}C{^{1}H} NMR (100 MHz, C_6D_6, 298 K): δ = 151.8 (d, $^3J_{CP}$ = 44 Hz, Cq-Ar^{Dipp}), 147.1 (m, Cq-Ar^{Dipp}), 139.9 (CH-PPh$_2$), 139.8 (CH-PPh$_2$), 139.3 (Cipso-Ar^{Dipp}), 138.9 (CH-PPh$_2$), 134.3 (d, CH-PPh$_2$), 131.7 (CH-PPh$_2$), 130.4 (CH-PPh$_2$), 127.2 (CH-PPh$_2$), 125.6 (CH-Ar^{Dipp}), 124.4 (CH-Ar^{Dipp}), 100.8 (m, C_5(CH$_3$)$_5$), 28.9 (CH-isopropyl), 27.8 (s, CH$_3$-isopropyl), 27.5 (CH-isopropyl), 26.7 (CH$_3$-isopropyl), 24.1 (CH$_3$-isopropyl), 22.8 (CH$_3$-isopropyl), 9.0 (C$_5$(CH$_3$)$_5$).

(κ^N:κ^P-$Ph_2PNDipp$)Cl_2Ge–IrClCp* (Compound 1b)

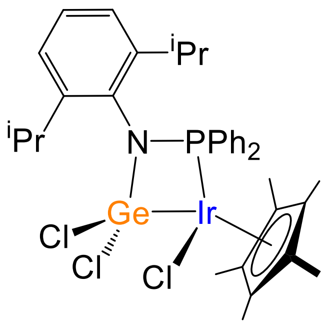

In a J. Young ampoule, $DippNHPPh_2$ (402 mg, 1.11 mmol) was dissolved in toluene (12 mL), reacted with ^nBuLi (0.44 mL, 1.11 mmol) at -78 ºC and stirred overnight. The solution was cooled at -78 ºC again and a suspension of $GeCl_2$·dioxane (386 mg, 1.66 mmol) in 10 mL of toluene was added dropwise and stirred about 2 hours. A solution of precursor $[IrCl_2Cp^*]_2$ (292 mg, 0.55 mmol) in 10 mL of toluene was added to the previous mixture and stirred overnight. The resulted mixture was filtered and solvent was removed. The solid obtained was washed with Et_2O (3 x 10 mL) and dried under vacuum to afford an orange-red solid. Crystals suitable for XRD were obtained by slow diffusion of pentane into the compound solution. Yield: 485 mg (50 %).

Anal. Calcd. for $C_{34}H_{42}GeIrCl_3NP$: C, 47.1; H, 4.9; N, 1.6. **Found**: C, 47.7; H, 5.4; N, 1.6.

^{1}H NMR (400 MHz, C_6D_6, 298 K): δ = 8.19 (br s, 1H, , CH-PPh_2), 7.54 (br s, 2H, CH-PPh_2*)*, 7.29 (br s, 1H, CH-PPh_2), 7.03-7.07 (m, 3H, CH-Ar^{Dipp}), 6.89 (s, 4H, CH-PPh_2), 6.68 (br s, 1H, CH-PPh_2), 6.44 (br s, 1H, CH-PPh_2), 4.37 (m, 1H, CH-isopropyl), 4.29 (m, 1H, CH-isopropyl), 1.45 (d, $^3J_{HH}$ = 6.7 Hz, 3H, CH_3-isopropyl), 1.42 (d, $^3J_{HH}$ = 6.6 Hz, 3H, CH_3-isopropyl), 1.30 (d, $^4J_{HP}$ = 3.1 Hz, 15H, Cp*), 0.52 (d, $^3J_{HH}$ = 6.8 Hz, 3H, CH_3-isopropyl), 0.28 (d, $^3J_{HH}$ = 6.6 Hz, 3H, CH_3-isopropyl).

^{31}P{^{1}H} NMR (160 MHz, C_6D_6, 298K): δ = 43.9 (s, Ir-PPh_2).

^{13}C{^{1}H} NMR (100 MHz, C_6D_6, 298 K): δ = 151.4 (Cq-Ar^{Dipp}), 147.7 (Cq-Ar^{Dipp}), 140.8 (d, $^2J_{CP}$ = 12 Hz , Cipso-Ar^{Dipp})), 134.4 (CH-PPh_2), 131.6 (CH-Ar^{Dipp}), 130.5 (CH-PPh_2), 128.0 (CH-PPh_2), 127.2 (CH-PPh_2), 127.0 (CH-PPh_2), 125.7 (CH-Ar^{Dipp}), 124.5 (CH-Ar^{Dipp}+CH-PPh_2), 95.3 ($C_5(CH_3)_5$), 28.8 (CH-isopropyl), 27.7 (CH_3-isopropyl), 27.5 (CH-isopropyl), 26.8 (CH_3-isopropyl), 24.0 (CH_3-isopropyl), 22.8 (CH_3-isopropyl), 8.5 ($C_5(CH_3)_5$).

[(κ^{Ge}:κ^{P}-Ph_2PNDippGeCl_2)(Rh(COD))$_2$(μ-Cl)] (Compound 2a)

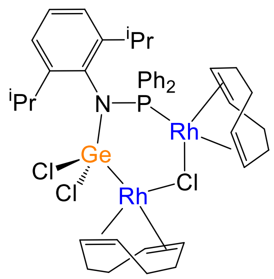

In a J. Young ampoule, DippNHPPh_2 (346 mg, 0.96 mmol) was dissolved in toluene (12 mL), reacted with ^{n}BuLi (0.42 mg, 1.05 mmol) at -78 ºC and stirred overnight. A suspension of GeCl_2·dioxane (301 mg, 1.3 mmol) in 10 mL of toluene was added dropwise and stirred about 2 hours. A solution of precursor [RhCl(COD)]$_2$ (414 mg, 1.05 mmol) in 10 mL of toluene was added to the previous mixture and stirred overnight. The resulted mixture was filtered and solvent was removed. The solid obtained was washed with Et_2O (3 x 10 mL) and dried under vacuum to afford an orange-red solid. Crystals suitable for XRD were obtained by slow diffusion of pentane into the compound solution. Yield: 678 mg (69 %).

Anal. Calcd. for $C_{40}H_{51}GeRh_2Cl_3NP$: C, 50.0; H, 5.4; N, 1.5. **Found**: C, 47.7; H, 5.3; N, 1.4.

^{1}H NMR (400 MHz, C_6D_6, 25 ºC): δ = 9.55 (t, $^{3}J_{HH}$ = 9.0 Hz, 1H, CH-PPh_2), 9.36 (dd, $^{3}J_{HH}$ = 14.2 Hz, 7.5 Hz, 1H, CH-PPh_2), 7.34 (t, $^{3}J_{HH}$ = 6.3 Hz, 1H, CH-PPh_2), 7.24 (t. $^{3}J_{HH}$ = 7.1 Hz, 1H, CH-PPh_2), 7.15 (m, 1H, CH-PPh_2), 7.03 (m, 3H, CH-Ar^{Dipp}), 6.91 (t, $^{3}J_{HH}$ = 7.1 Hz, 1H, CH-PPh_2), 6.83 (br s, 1H, CH-COD), 6.67 (m, 2H, CH-PPh_2), 6.45 (t, $^{3}J_{HH}$ = 7.0 Hz, 1H, CH-PPh_2), 6.31 (t, $^{3}J_{HH}$ = 8.3 Hz, 1H, CH-PPh_2), 5.37 (br s, 1H, CH-COD), 5.26 (t, $^{3}J_{HH}$ = 6.8 Hz, 1H, CH-COD), 5.20 (br s, 1H, CH-COD), 5.12 (br s, 1H, CH-COD), 4.47 (q, $^{3}J_{HH}$ = 7.4 Hz, 1H, CH-COD), 4.32 (br s, 1H, CH-COD), 4.19 (br s, 1H, CH-COD), 4.12 (m, 1H, CH-isopropyl), 3.76 (dq, $^{3}J_{HH}$ = 13.0, 6.5 Hz, 1H, CH-isopropyl), 2.74 (m, 3H, CH_2-COD), 2.48 (dq, $^{3}J_{HH}$ = 16.5, 10.1 H, 1H, CH_2-COD), 2.36 (dt, $^{3}J_{HH}$ = 16.7, 8.4 Hz, 1H, CH_2-COD), 2.07 (m, 1H, CH_2-COD), 1.91 (m, 4H, CH_2-COD), 1.75 (m, 2H, CH_2-COD), 1.64 (two d, 6H, CH_3-isopropyl), 1.46 (br s, 1H, CH_2-COD), 1.33 (two d, $^{3}J_{HH}$ = 6.6, 8.3 Hz, 3H, CH_2-COD), 0.43 (d, $^{3}J_{HH}$ = 6.6 Hz, 3H, CH_3-isopropyl) 0.29 (d, $^{3}J_{HH}$ = 6.7 Hz, 3H, CH_3-isopropyl).

^{31}P{^{1}H} NMR (160 MHz, C_6D_6, 25 ºC): δ = 63.7 (d, $^{1}J_{PRh}$ = 152 Hz, Rh-PPh_2).

^{13}C{^{1}H} NMR (100 MHz, C_6D_6, 25 ºC): δ = 150.2 (Cq-Ar^{Dipp}), 149.1 (Cq-Ar^{Dipp}), 141.1 (d, $^{2}J_{CP}$ = 28 Hz, Cipso-Ar^{Dipp}), 136.8 (d, $^{3}J_{CP}$ = 7 Hz, CH-PPh_2), 136.6 (CH-PPh_2), 133.2 (d, $^{3}J_{CP}$ = 6 Hz, CH-PPh_2), 131.7 (CH-Ar^{Dipp}), 130.4 (CH-PPh_2), 128.7 (CH-Ar^{Dipp}), 128.5

(CH-PPh_2), 128.2 (s, CH-PPh_2), 127.7 (s, CH-PPh_2), 125.6 (s, CH-PPh_2), 125.2 (s, CH-Ar^{Dipp}), 108.4 (m, CH-COD), 107.6 (m, CH-COD), 100.3 (d, $^1J_{CRh}$ = 8 Hz, CH-COD), 94.6 (m, CH-COD), 79.1 (d, $^1J_{CRh}$ = 14 Hz, CH-COD), 76.9 (d, $^1J_{CRh}$ = 13 Hz, CH-COD), 75.8 (d, $^1J_{CRh}$ = 13 Hz, CH-COD), 75.2 (d, $^1J_{CRh}$ = 15 Hz, CH-COD), 36.9 (CH_2-COD), 33.9 (CH_2-COD), 33.4 (CH_2-COD), 32.3 (CH_2-COD), 31.5 (CH_2-COD), 30.2 (CH_2 COD), 29.6 (CH-isopropyl), 28.5 (CH-isopropyl), 28.4 (CH_3-isopropyl), 27.0 (CH_2 COD), 26.7 (CH_3-isopropyl), 25.6 (CH_3-isopropyl), 22.6 (CH_3-isopropyl).

[(κ^{Ge}:κ^{P}-$Ph_2PNDippGeCl_2$)(Ir(COD))$_2$(μ-Cl)] (Compound 2b)

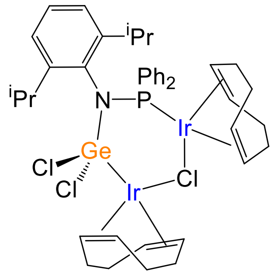

In a J. Young ampoule, $DippNHPPh_2$ (356 mg, 0.98 mmol) was dissolved in toluene (12 mL), reacted with ^nBuLi (0.43 mL, 1.08 mmol) at -78 ºC and stirred overnight. The solution was cooled at -78 ºC again and a suspension of $GeCl_2$·dioxane (301 mg, 1.30 mmol) in 10 mL of toluene was added dropwise and stirred about 2 hours. A solution of precursor $[IrCl(COD)]_2$ (545 mg, 0.8 mmol) in 10 mL of toluene was added to the previous mixture and stirred overnight. The resulted mixture was filtered and solvent was removed. The solid obtained was washed with Et_2O (3 x 10 mL) and dried under vacuum to afford an orange-red solid. Crystals suitable for XRD were obtained by slow diffusion of pentane into the compound solution. Yield: 635 mg (54 %).

Anal. Calcd. for $C_{44}H_{63}GeIr_2Cl_3NP$: C, 44.0; H, 5.3; N, 1.2. **Found**: C, 44.2; H, 5.0; N, 1.5.

^{1}H NMR (400 MHz, C_6D_6, 25 ºC): δ = 9.57 (t, $^3J_{HH}$ = 10.3 Hz, $^3J_{HH}$ = 9.3 Hz, 1H, CH-PPh_2), 9.04 (dd, $^3J_{HH}$ = 14.0 Hz, 7.5 Hz, 1H, CH-PPh_2), 7.27 (m, 2H, CH-PPh_2), 6.99 (m, 3H, CH-Ar^{Dipp}), 6.91 (m, 2H, CH-PPh_2), 6.69 (dt, $^3J_{HH}$ = 15.0 Hz, $^3J_{HH}$ = 7.3 Hz, 2H, CH-PPh_2), 6.51 (t, $^3J_{HH}$ = 7.8 Hz, 1H, CH-PPh_2), 6.34 (t, $^3J_{HH}$ = 8.9 Hz, 1H, CH-PPh_2), 5.08 (t, $^3J_{HH}$ = 7.1, 1H, CH-COD), 5.00 (t, $^3J_{HH}$ = 6.6, 1H, CH-COD), 4.91 (q, $^3J_{HH}$ = 7.3, 1H, CH-COD), 4.79 (br s, 2H, CH-COD), 4.28 (br s, 1H, CH-COD), 4.20 (p, $^3J_{HH}$ = 6.7 Hz, 1H, CH-isopropyl), 4.05 (q, $^3J_{HH}$ = 7.6 Hz, 1H, CH-COD), 3.80 (br s, 1H, CH-COD), 3.58 (q, $^3J_{HH}$ = 6.7 Hz, 1H, CH-isopropyl), 2.33 (m, 1H, CH_2-COD), 2.06-2.26 (m, 2H, CH_2- COD), 1.97 (br s, 1H, CH_2-COD), 1.69-1.93 (m, 4H, CH_2-COD), 1.66 (d, $^3J_{HH}$ = 6.6 Hz, 3H, CH_3-isopropyl), 1.61 (br s, 1H, CH_2-COD), 1.54 (m, 1H, CH_2-COD), 1.43 (d, $^3J_{HH}$ = 6.6 Hz, 3H, CH_3-isopropyl), 1.29 (m, 2H, CH_2-COD), 1.20 (d, $^3J_{HH}$ = 8.1 Hz, 1H, CH_2-COD), 1.12 (t, $^3J_{HH}$ = 7.0 Hz, 2H, CH_2-COD), 1.00 (m, 1H, CH_2-COD), 0.48 (d, $^3J_{HH}$ = 6.6 Hz, 3H, CH_3-isopropyl), 0.23 (d, $^3J_{HH}$ = 6.6 Hz, 3H, CH_3-isopropyl).

$^{31}P\{^1H\}$ NMR (160 MHz, C_6D_6, 25 ºC): δ = 48.9 (s, Ir-PPh_2).

$^{13}C\{^1H\}$ NMR (100 MHz, C_6D_6, 25 ºC): δ = 149.6 (Cq-Ar^{Dipp}), 148.6 (Cq-Ar^{Dipp}), 141.5 (m, CH-PPh_2), 136.4 (Cipso-Ar^{Dipp}), 135.7 (d, $^3J_{CP}$ = 13 Hz, CH-PPh_2), 133.4 (m, CH-PPh_2), 131.2 (CH-Ar^{Dipp}), 130.2 (CH-PPh_2), 127.9 (CH-Ar^{Dipp}), 127.4 (d, $^3J_{CP}$ = 10 Hz, CH-PPh_2), 127.1 (d, $^3J_{CP}$ = 9 Hz, CH-PPh_2), 124.7 (CH-Ar^{Dipp}), 86.5 (CH-COD), 77.3 (CH-COD), 62.6 (CH-COD), 62.1 (CH-COD), 59.4 (CH-COD), 37.9 (CH_2-COD), 34.8 (CH_2-COD), 34.0 (CH_2-COD), 31.9 (CH_2-COD), 30.1 (CH_2-COD), 29.7 (CH_2-COD), 29.2 (CH-isopropyl+ CH_2-COD), 27.8 (CH_3-isopropyl), 27.1 (CH_3-isopropyl), 26.4 (CH_2-COD), 26.1 (CH_3-isopropilo), 24.9 (CH_3-isopropyl), 22.1 (CH_3-isopropyl).

3. NMR spectra of compounds

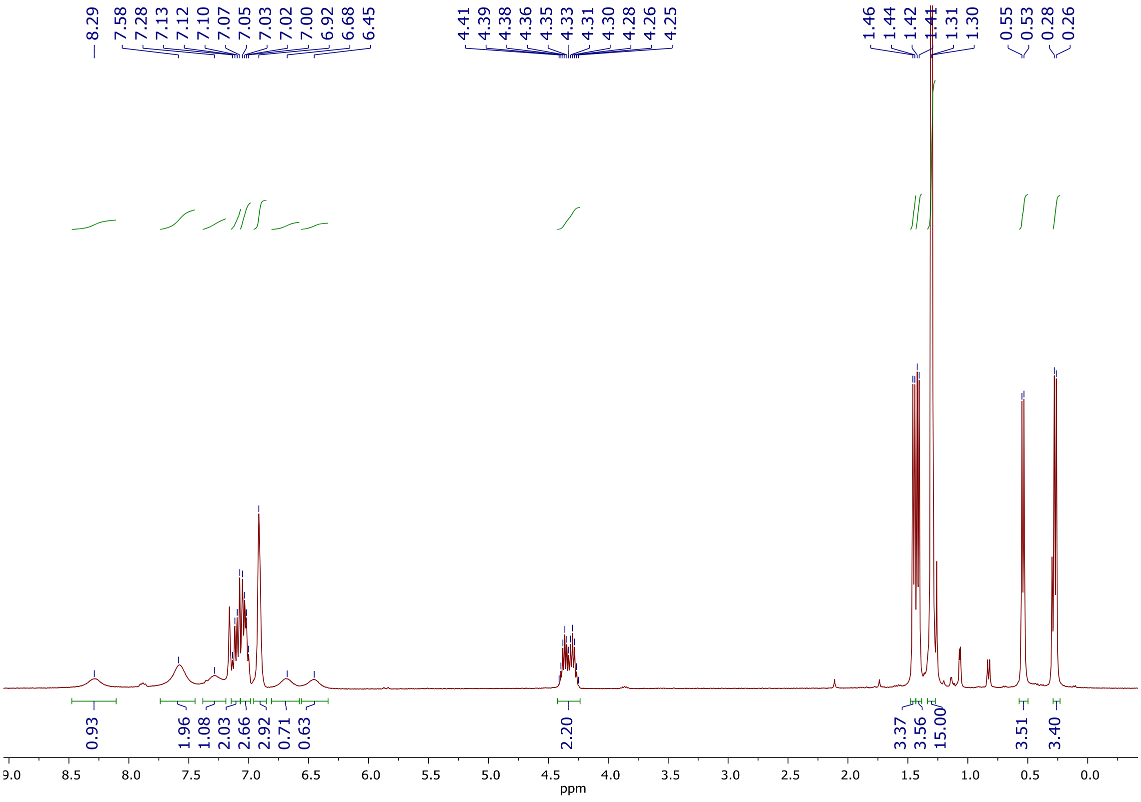

Figure S1. ^{1}H NMR (400 MHz, C_6D_6, 25 ºC) for compound **1a**.

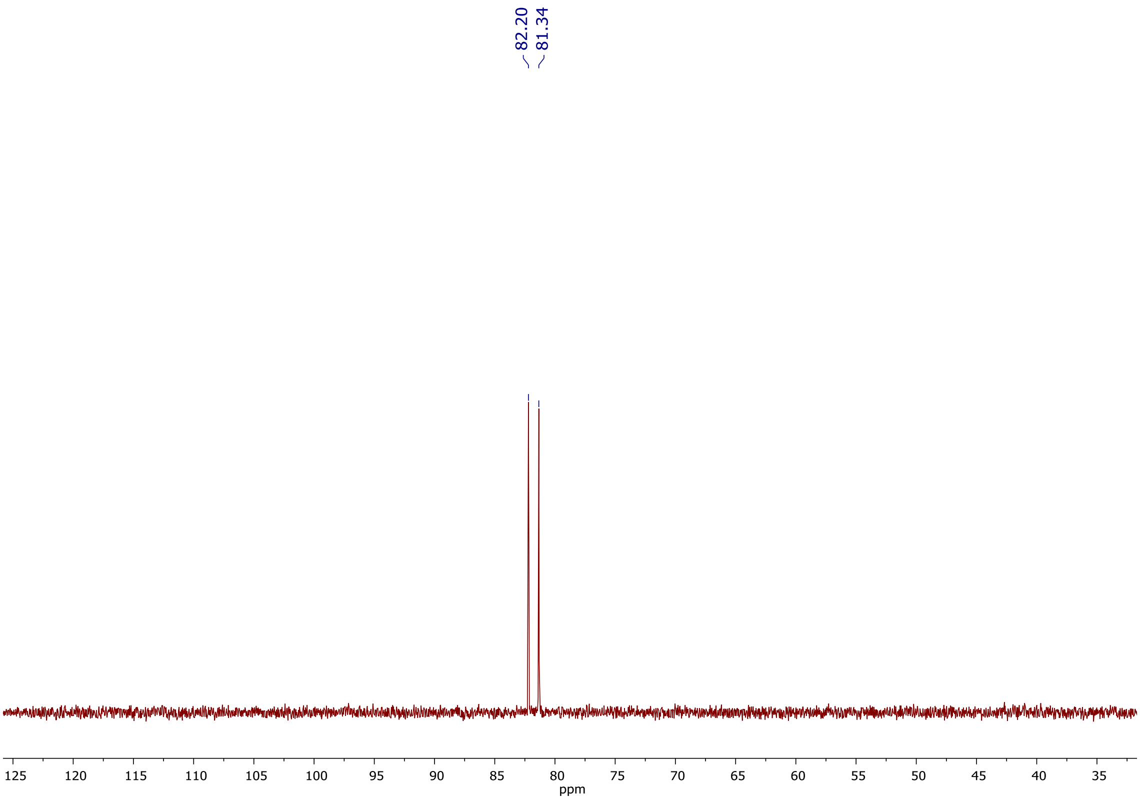

Figure S2. $^{31}P\{^1H\}$ NMR (160 MHz, C_6D_6, 25 ºC) for compound **1a**.

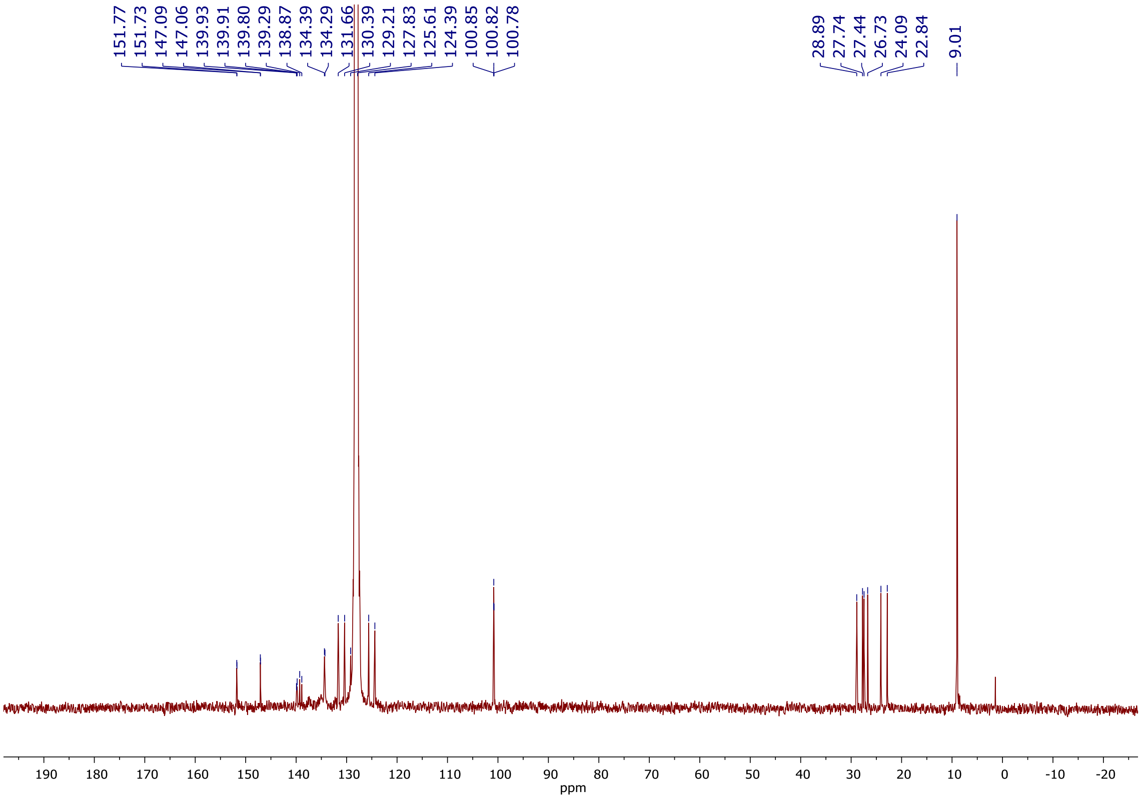

Figure S3. $^{13}C\{^{1}H\}$ NMR (100 MHz, C_6D_6, 25 ºC) for compound **1a**.

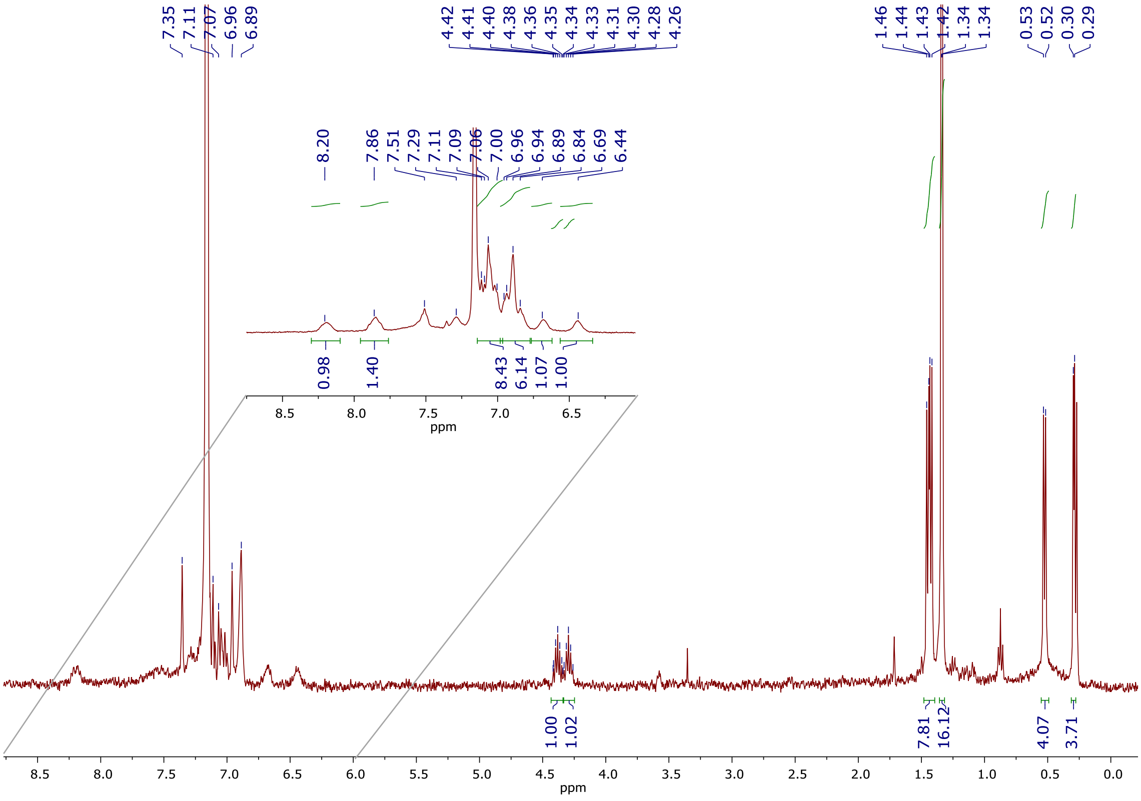

Figure S4. ^{1}H NMR (400 MHz, C_6D_6, 25 ºC) for compound **1b**.

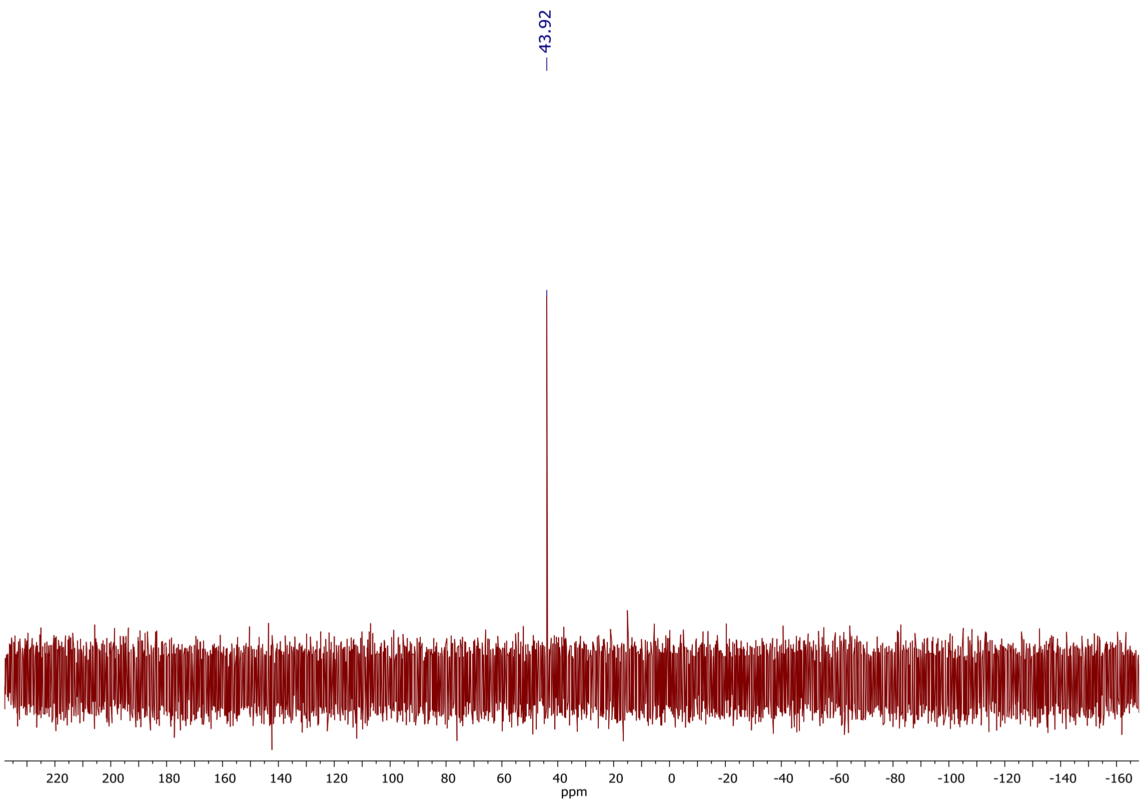

Figure S5. $^{31}P\{^{1}H\}$ NMR (160 MHz, C_6D_6, 25 ºC) for compound **1b**.

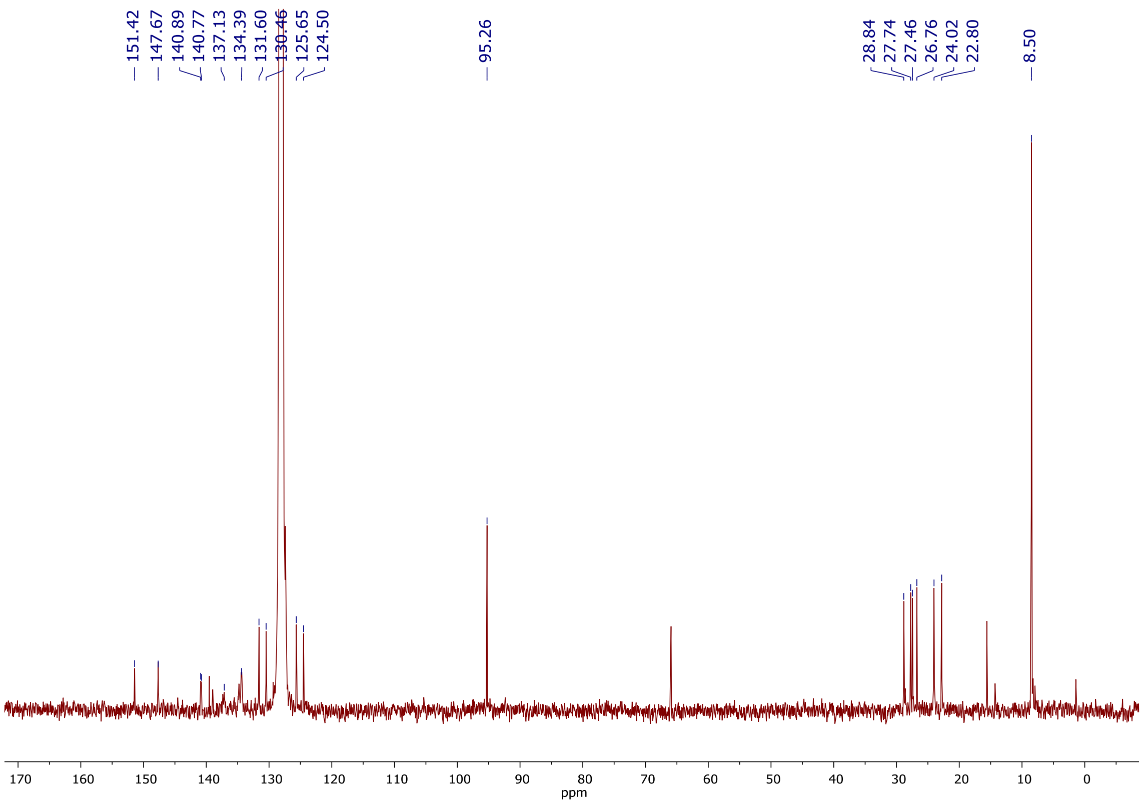

Figure S6. $^{13}C\{^{1}H\}$ NMR (100 MHz, C_6D_6, 25 ºC) for compound **1b**.

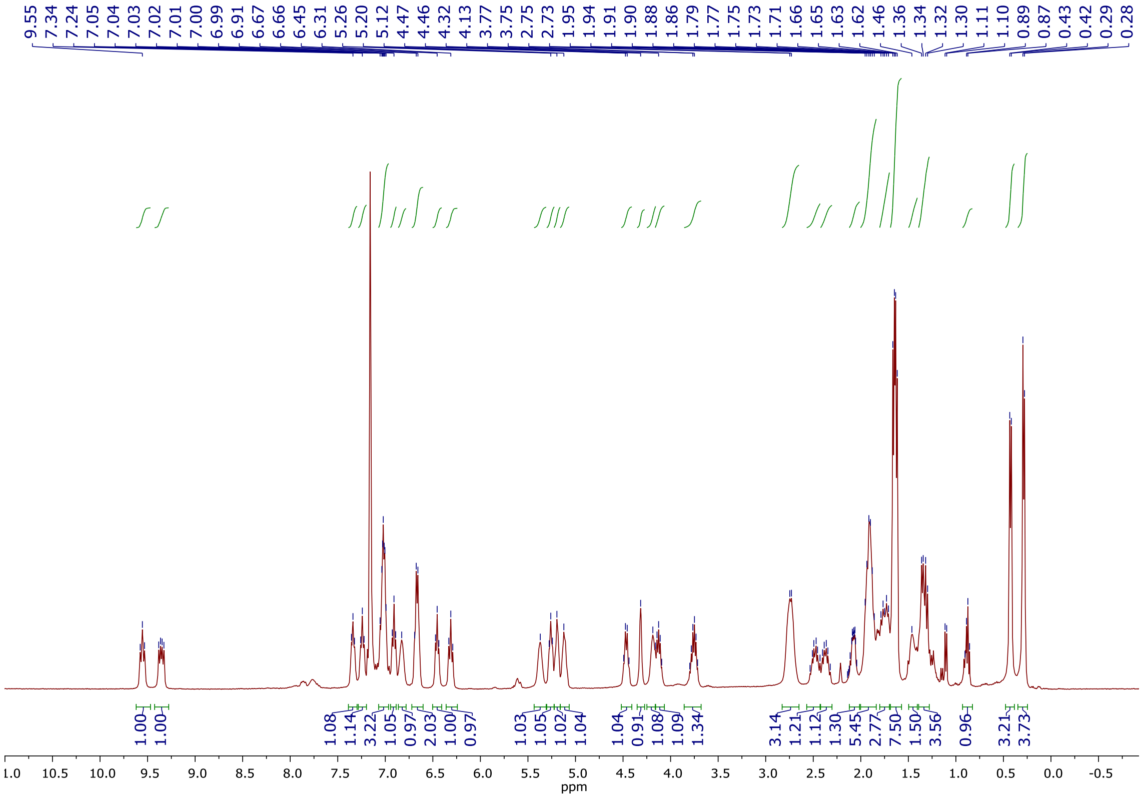

Figure S7. ^{1}H NMR (400 MHz, C_6D_6, 25 ºC) for compound **2a**.

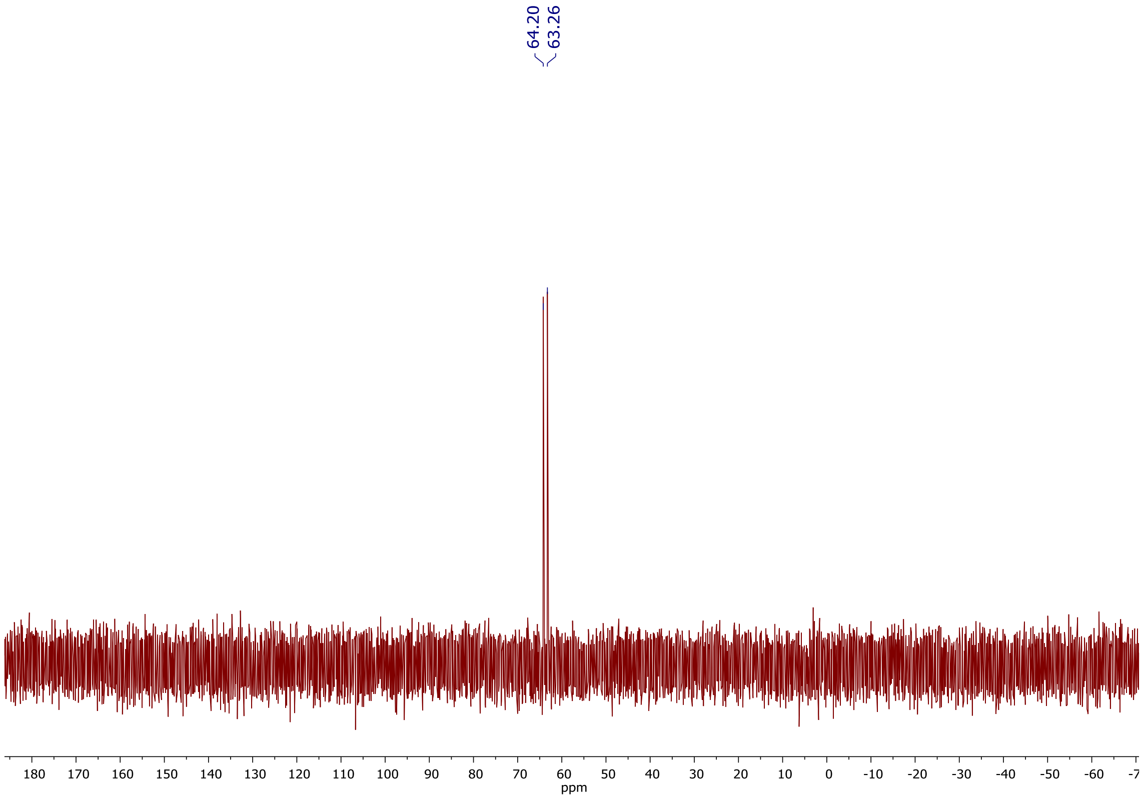

Figure S8. $^{31}P\{^{1}H\}$ NMR (160 MHz, C_6D_6, 25 ºC) for compound **2a**.

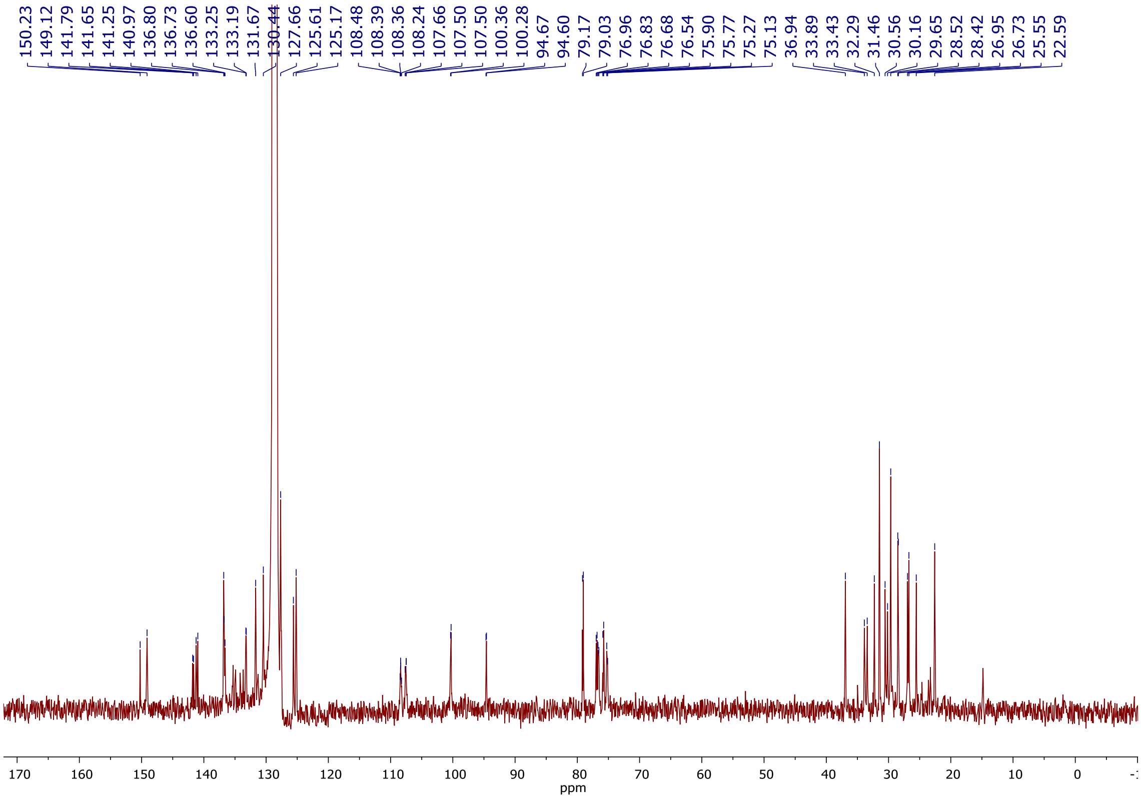

Figure S9. $^{13}C\{^1H\}$ NMR (100 MHz, C_6D_6, 25 ºC) for compound **2a**.

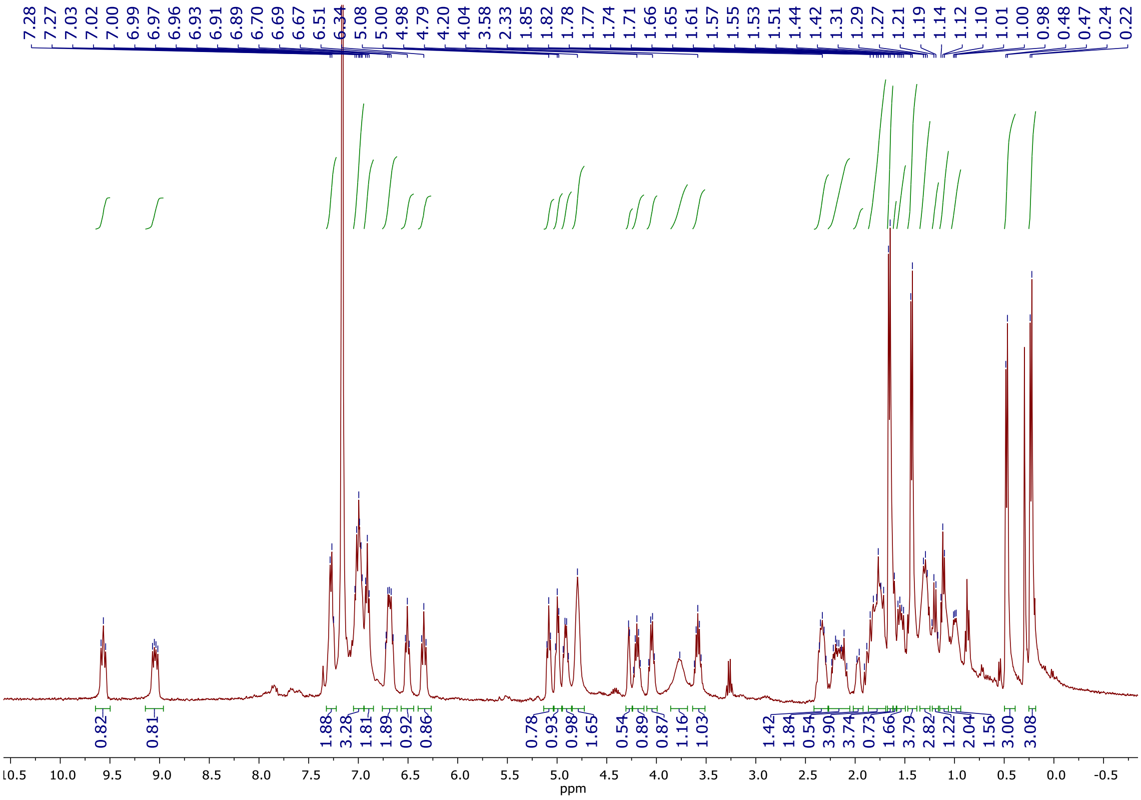

Figure S10. ^1H NMR (400 MHz, C_6D_6, 25 ºC) for compound **2b**.

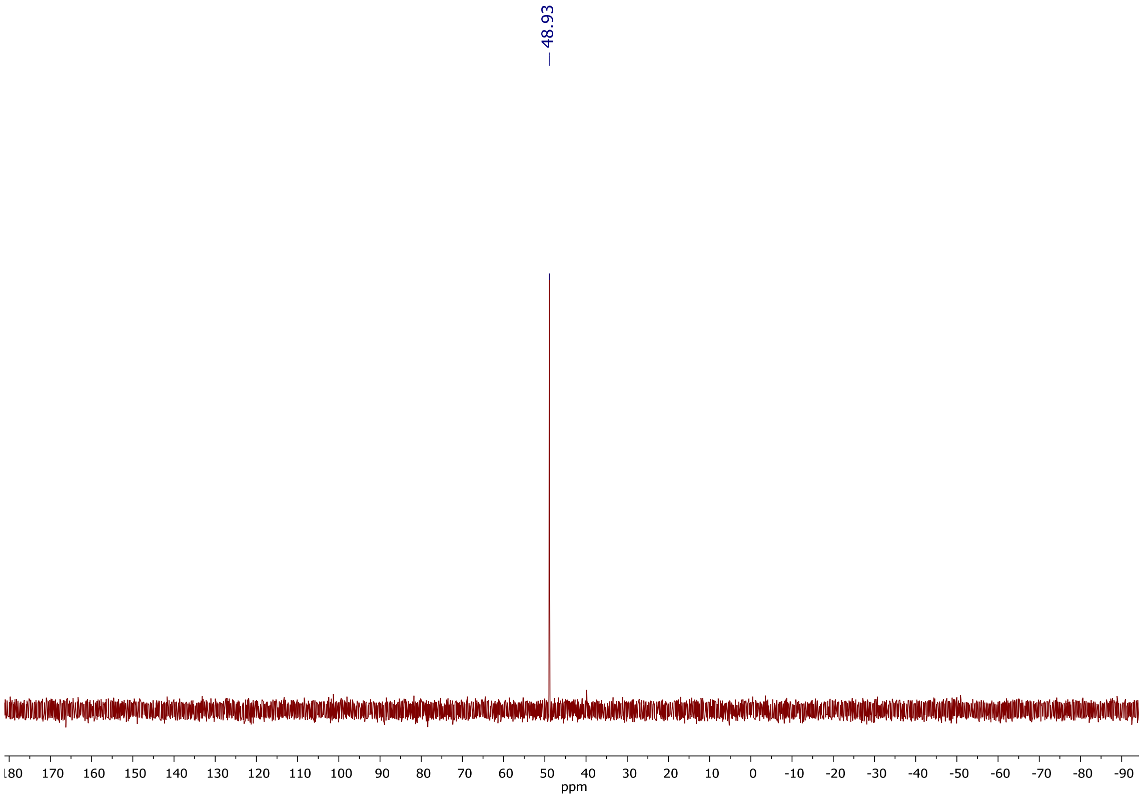

Figure S11. $^{31}P\{^{1}H\}$ NMR (160 MHz, C_6D_6, 25 ºC) for compound **2b**.

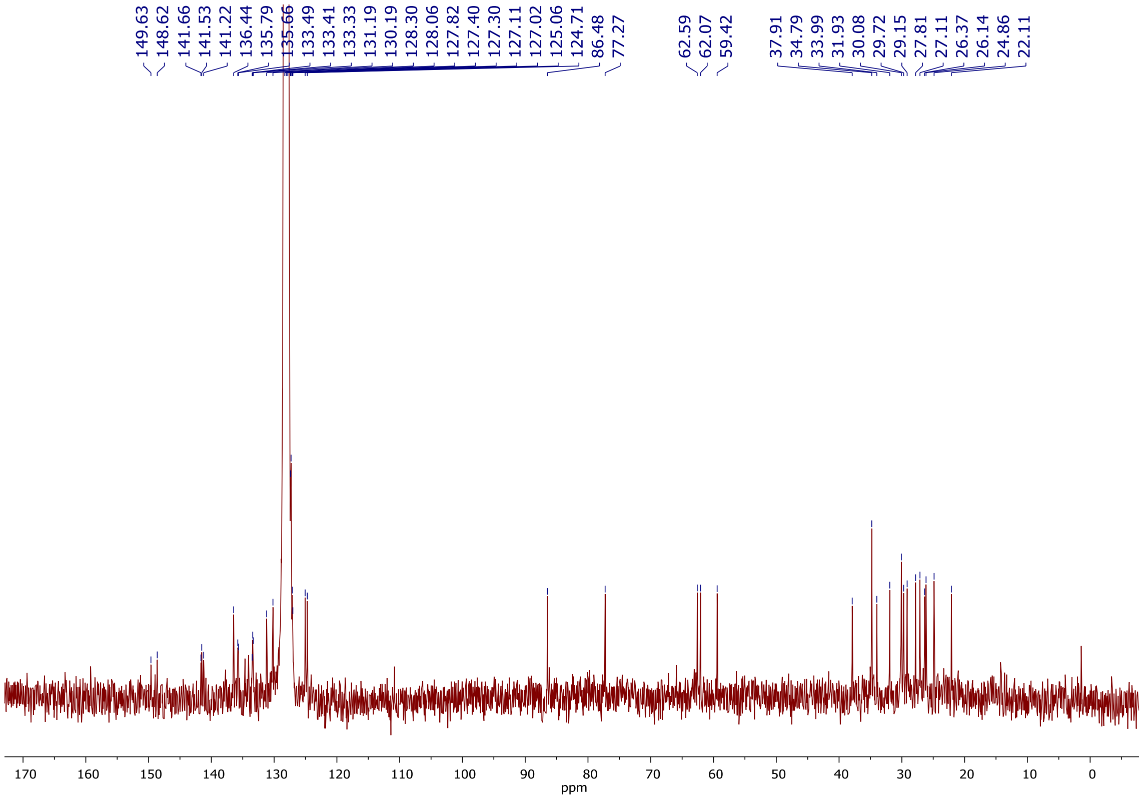

Figure S12. $^{13}C\{^{1}H\}$ NMR (100 MHz, C_6D_6, 25 ºC) for compound **2b.**

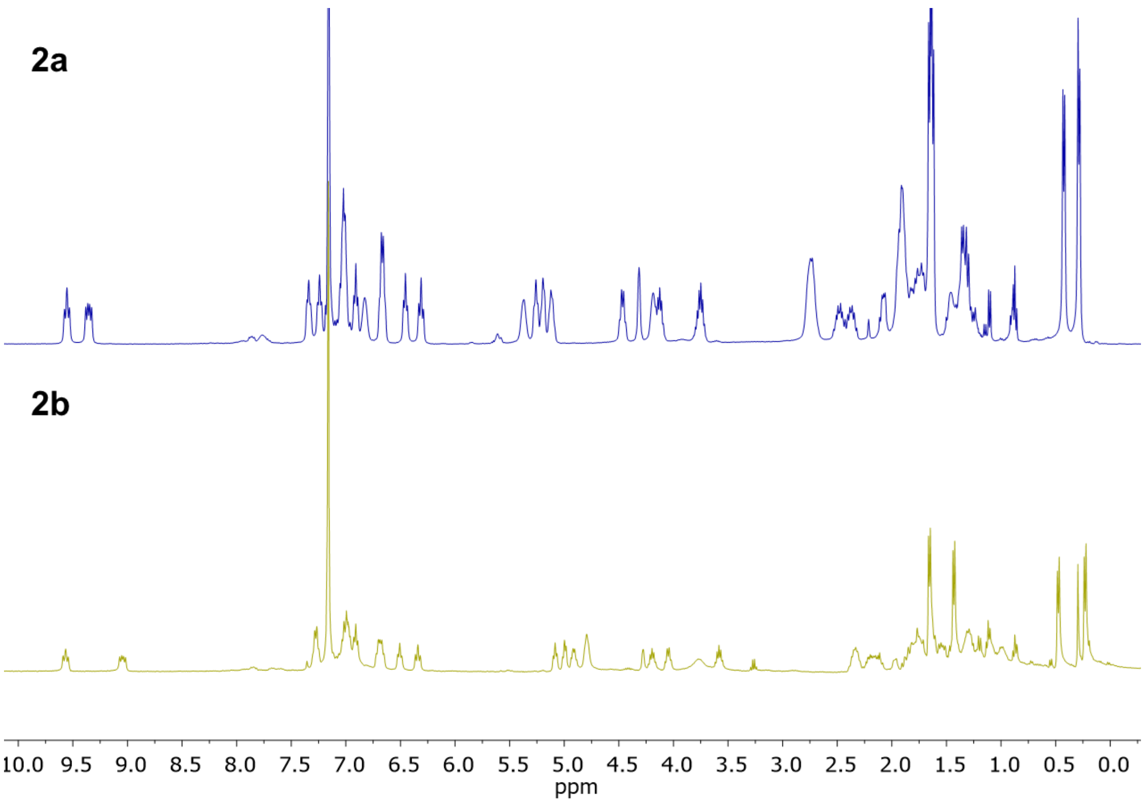

Figure S13. Comparison of ^{1}H NMR spectra for species **2a** and **2b**

4. Crystal structure determinations

Low-temperature diffraction data were collected on a D8 Quest APEX-III single crystal diffractometer with a Photon III detector and a IμS 3.0 microfocus X-ray source. Data were collected by means of ω and φ scans using monochromatic radiation λ(Mo Kα1) = 0.71073 Å. The structures were solved with SHELXT and was refined against F^2 on all data by full-matrix least squares with SHELXL, using Olex2 as graphical interface. All non-hydrogen atoms were refined anisotropically. Hydrogen atoms were included in the model at geometrically calculated positions and refined using a riding model, unless otherwise noted. A summary of the fundamental crystal and refinement data are given in Table S1. Atomic coordinates, anisotropic displacement parameters and bond lengths and angles can be found in the cif files, which have been deposited in the Cambridge Crystallographic Data Centre with no. CCDC 2491486-2491488. These data can be obtained free of charge from The Cambridge Crystallographic Data Centre via www.ccdc.cam.ac.uk/data_request/cif

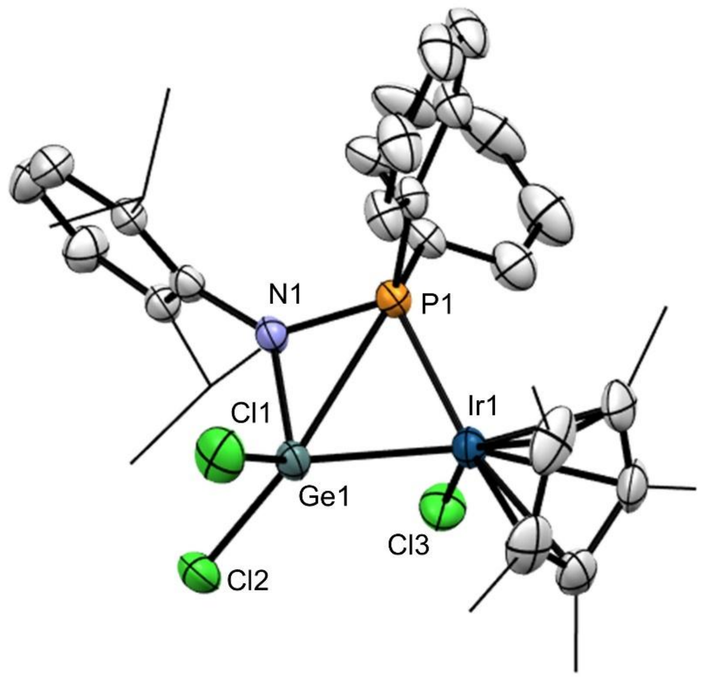

Figure S14. ORTEP diagram of complex **1b**. Hydrogen atoms have been excluded and isopropyl groups and methyl termini of the Cp* ligands are represented in wireframe format for clarity. Thermal ellipsoids are set at 50% probability.

Table S1. Crystal data and structure refinement for compounds **1a, 1b** and **2a.**

	Compound **1a**	Compound **1b**	Compound **2a**
Empirical formula	$C_{34}H_{42}Cl_3GeNPRh$	$C_{34}H_{42}Cl_3GeIrNP$	$C_{40}H_{51}Cl_3GeNPRh_2$
Formula weight	777.50	866.79	961.54
Temperature/K	296.15	193.00	193
Crystal system	monoclinic	monoclinic	monoclinic
Space group	$P2_1/n$	$P2_1/n$	$P2_1/c$
a/Å	12.2918(9)	12.3450(10)	19.2777(19)
b/Å	16.6438(10)	16.5276(11)	9.9038(8)
c/Å	17.0098(10)	17.0978(12)	20.603(2)
α/°	90	90	90
β/°	101.328(2)	101.409(3)	98.378(4)
γ/°	90	90	90
Volume/Å^3	3412.1(4)	3419.6(4)	3891.6(7)
Z	4	4	4
ρ_{calc}g/cm^3	1.514	1.684	1.641
μ/mm^{-1}	1.671	5.072	1.883
F(000)	1584.0	1712.0	1944.0
Crystal size/mm^3	0.18 × 0.15 × 0.1	0.2 × 0.08 × 0.07	0.1 × 0.1 × 0.07
Radiation	MoKα (λ = 0.71073)	MoKα (λ = 0.71073)	MoKα (λ = 0.71073)
2Θ range for data	5.464 to 50.496	4.172 to 51.996	3.996 to 54.998
Index ranges	-14 ≤ h ≤ 14, -19	-15 ≤ h ≤ 15, -20	-25 ≤ h ≤ 25, -12 ≤
Reflections collected	41706	87405	26902
Independent reflections	6171 [R_{int} = 0.0477, R_{sigma} = 0.0338]	6728 [R_{int} = 0.0932, R_{sigma} = 0.0349]	8919 [R_{int} = 0.0854, R_{sigma} = 0.1262]
Data/restraints/parameters	6171/0/379	6728/0/379	8919/0/431
Goodness-of-fit on F^2	1.024	1.356	1.185
Final R indexes [I>=2σ (I)]	R_1 = 0.0350, wR_2 = 0.0807	R_1 = 0.0375, wR_2 = 0.0803	R_1 = 0.0853, wR_2 = 0.1116
Final R indexes [all data]	R_1 = 0.0502, wR_2 = 0.0881	R_1 = 0.0690, wR_2 = 0.1081	R_1 = 0.1463, wR_2 = 0.1256
Largest diff. peak/hole / e	1.43/-0.60	2.90/-1.40	1.90/-1.24
CCDC number	2491486	2491488	2491487

5. Computational studies

Geometry optimization of minima and transition states were carried out using the Gaussian software package.[5] Optimizations were performed without symmetry restrictions using DFT methods. The B3PW91 functional[6] was used, with empirical dispersion accounted for by adding Grimme's D3 version with Becke-Johnson damping.[7] The def2-TZVP basis set[8] was applied to all atoms in the molecules, with the inner electrons of the transition metal ions being accounted for by the corresponding Stuttgart-Dresden ECPs, SDD.[9] Bulk solvent effects (toluene) were included during optimization with the SMD continuum model.[10] The extended wavefunction, .wfx, and NBO .47 files were calculated on previously optimized geometries, using the triple-ζ basis set def2-TZVP basis set for all atoms, which includes and ECP for Ir.[11] NBO analysis was performed with the NBO6.0[11] software.

We carried out computational analysis to shed light on the bonding of the phosphinoamido chlorogermyl moiety with the transition metals in our complexes. Geometry optimizations at the DFT-B3PW91-D3BJ/def2-TZVP+SDD level of theory reproduced well the solid-state molecular geometries of complexes **1a**, **1b** and **2a**, obtained by XRD analysis. In species **1** [PNGeMCl(η^5-C_5Me_5)] (**1a** M = Rh; **1b**, M = Ir), the TM-Ge and TM-P bond distances present values of 2.3798 and 2.2833 Å for **1a** and 2.400 and 2.2855 Å for compound **1b**. The same tendency is observed for the P-MT-Ge angle. Both species yield values similar to the experimental ones: 69.63 º (**1a**) and 69.29 º (**1b**). Natural Bond Orbital (NBO) analysis of these species revealed Wiberg Bond Indices (WBI) for the Rh-Ge and Ir-Ge bonds of 0.47 and 0.53, respectively, which are similar to those of the TM-P bonds (0.53 and 0.60). Focusing on the transition metal ions of **1a** and **1b**, the NBO analyses localized three d lone pairs on each atom, as expected for Rh(III) and Ir(III) ions, which have 6 d valence electrons. In addition, the Rh-Ge bond of **1a** is described in terms of a largely covalent σ interaction between a sd orbital of rhodium (40%) and a s^3p orbital of germanium (60%). While the germanium atom has three lone vacancies (LV), or vacant orbitals capable of accepting electron density from other atoms, the NBO analysis did not locate a π back-donation component to the Rh-Ge bond, instead these LVs, one with sp^3 and two with p character, receive electron density from the neighboring N and Cl atoms, effectively taking part in the formation of σ Ge-E (dative) bonds with these. With the Natural Localized Molecular Orbitals (NLMO) we confirm these dative bonds as all three lone pairs (LP) present an important contribution of Germanium. The Rh-P bond is characterized as a sigma interaction involving an sd^2

orbital of Rh (40%) and an sp^3 orbital from P (60%). Table S2 summarizes this information. The NBO analysis of the Ir-Ge bond of **1b**, reveals a very similar bonding picture: a σ bond is established between the two atoms involving an sd^2 orbital of iridium (47%) and a sp orbital of germanium (53%). As with the rhodium species, no π back-donation component was identified. The Ir-P bond presents again a σ interaction between a sd^2 orbital of iridium (40%) and a sp^3 orbital of phosphorous (60%). The germanium environment in this case is described as one Ge-Cl bond (24% of sp^3 on Ge and 76% sp^3 on Cl) and two p vacant orbitals (LV) which are accepting electron density from another chloride and nitrogen atoms (See Table S3).

For compound **2a**, four d lone pairs on each atom are localized, resulting in Rh(I) centers. A WBI of 0.52 is obtained between the germanium and the bonded rhodium atom. This value is analogous to that obtained for the Rh-P interaction (0.55), once again emphasizing their similarity as ligands. According to this, a σ interaction between Ge and Rh is observed, showing 61% contribution of an s^2p bond from germanium and 39% from an sd^3 orbital of rhodium. The Rh-P bond shows an interaction between a p orbital of rhodium (38%) and an sp^2 orbital of phosphorous (62%). For the distant rhodium, a WBI of 0.044 is found. The remaining bonds associated with the Ge atom show typical WBI values of sigma interactions, similar to those seen in the other compounds (0.499 for Ge-N; 0.685 and 0.729 for Ge-Cl). In this case, the analysis reveals one Ge-Cl sigma bond with a 22% of a sp^3 orbital of Ge and 78% of an sp3 orbital of Cl. The remaining sigma bonds are described interactions between lone vacancy orbitals on Ge and lone pairs from Cl and N, as we shown for species **1b** (Table S4). From this analysis, it can be concluded that this species features a germyl ligand and two Rh(I) atoms, one of which forms a σ bond with Ge and the other with P. There is no direct electronic interaction between Rh atoms; they are connected by a bridging Cl atom.

The same analysis was performed for compound **2b**. A species with two transition metal atoms and a Ge-Ir bond was also obtained. This interaction exhibits a WBI of 0.57, similar to the other bonds involving Ge. For the chloride atoms, WBIs of 0.74 and 0.70 were found, while the Ge-N bond has a value of 0.51. The iridium atoms are also connected via a bridging chloride, which has a WBI of 0.36 with each iridium atom. The Ir-P interaction shows similarities to that observed in species **2a**, with a WBI of 0.59. Looking more closely at the NBO analysis, the Ir-Ge bond is defined as a σ interaction between an sp orbital of Ge (65%) and an sd orbital of Ir (35%). Its NLMO corresponds to 92.8% of

the initial NBO, indicating minimal delocalization. For the Ge atom, interactions are described as two σ bonds with the chloride atoms and a donation from a lone pair of nitrogen to the empty p orbital on Ge. The bond with nitrogen is described as delocalization from an sp^3 LP of N over a Ge p orbital. The NLMO analysis shows a contribution of 14.4% from Ge. Turning our attention to the iridium atoms, as expected, Ir-P interaction is very similar to the Ir-Ge one, consistent with the previous three compounds. The analysis indicates a σ bond between a sd orbital of iridium (32%) and an sp^2 orbital of phosphorous (68%). The bridging Cl presents a p orbital delocalized over both iridium atoms, with a contribution of 4% to the NLMO of each Ir. The other sp^2 orbital shows contributions of 6.5% from the metal centers (Table S5).

Upon analyzing the four species through a comprehensive study of the bonding interactions via Natural Bond Orbital (NBO) analysis, the robustness of these systems was confirmed.

Table S2. Relevant NBO results including major donor-acceptor interactions for compound **1a**.

Entry	Donor NBO (composition) occupancy (e^-)	Acceptor NBO / occupancy	ΔE_{ij} kcal·mol^{-1}	NLMO %parent NBO// composition %
1	[a]LP Cl (sp^3) / 1.53	LV Ge (sp^3) / 0.59	143.90	72.5 / 72.5 Cl, 24.4 Ge, 1.1 Rh,
2	LP Cl (sp^3) / 1.58	LV Ge (p) / 0.45	101.16	76.4 / 76.4 Cl, 19.5 Ge, 2.6 Cl
3	LP N (sp3) / 1.66	LV Ge (p) / 0.36	125.0	82.1 / 82.1 C, 14.0 Ge, 0.7 Cl
4	[b]BD σ 0.72 C_{33} (p) + 0.69 C_{34} (p) / 1.57	[c]BD σ* 0.78 Rh (sd) – 0.63 Ge (s^3p) / 0.49	50.6	76.0 / 40.7 C_{33}, 35.4 C_{34}, 8.0 Rh, 6.8 Ge
5	BD σ 0.63 Rh (sd) + 0.78 Ge (s^3p) / 1.78			
6	BD σ 0.63 Rh (sd^2) + 0.77 P (sp^3) / 1.85			

Table S3. Relevant NBO results including major donor-acceptor interactions for compound **1b**.

Entry	Donor NBO (composition) occupancy (e^-)	Acceptor NBO / occupancy	ΔE_{ij} kcal·mol^{-1}	NLMO %parent NBO// composition %
1	LP Cl (sp^3) / 1.57	LV Ge (p) / 0.50	181.9	76.8 / 76.8 Cl, 21.2 Ge, 0.6 Ir
2	LP N (sp3) / 1.66	LV Ge (p) / 0.37	132.3	82.1 / 82.0 N, 14.4 Ge, 0.8 Cl
3	BD σ 0.73 C_{32} (p) + 0.68 C_{33} (p) / 1.56	[c]BD σ* 0.70 Ge (sp) – 0.72 Ir (sd2) / 0.5	104.3	76.0 / 42.1 C_{32}, 33.9 C_{33}, 7.3 Ge, 8.4 Ir

4	BD σ 0.49 Ge (sp3) + 0.87 Cl (sp3) / 1.98			
5	BD σ 0.69 Ir (sd2) + 0.72 Ge (sp) / 1.86			
6	[b]BD σ 0.63 Rh (sd^2) + 0.77 P (sp^3) / 1.87			

Table S4. Relevant NBO results including major donor-acceptor interactions for compound **2a**.

Entry	Donor NBO (composition) occupancy (e^-)	Acceptor NBO / occupancy	ΔE_{ij} kcal·mol^{-1}	NLMO %parent NBO// composition %
1	LP Cl (sp^3) / 1.60	LV Ge (p) / 0.48	148.9	78.4 / 78.4 Cl, 19.8 Ge, 0.6 Rh
2	LP N (sp3) / 1.66	LV Ge (p) / 0.37	119.3	82.1 / 82.2 N, 13.8 Ge, 0.8 Cl
3	LP Cl (sp3) / 1.65	LV Rh_2 (s) / 0.41	38.5	82.0 / 82.0 Cl, 5.6 Rh_2, 6.2 Rh_3, 1.1 Ge
4		LV Rh_3 (s) / 0.42	38.1	
5	LP Cl (p) / 1.76	LV Rh_2 (s) / 0.41	25.7	87.8 / 87.8 Cl, 3.8 Rh_2, 3.4 Rh_3, 0.7 P
6		LV Rh_3 (s) / 0.42	23.5	
8	BD σ 0.78 Ge (s2p) + 0.63 Rh (sd3) / 1.93			
9	BD σ 0.47 Ge (sp3) + 0.88 Cl (sp3) / 1.98			
10	BD σ 0.62 Rh (sd^3) + 0.79 P (sp^2) / 1.92			

Table S5. Relevant NBO results including major donor-acceptor interactions for compound **2b**.

Entry	Donor NBO (composition) occupancy (e^-)	Acceptor NBO / occupancy	ΔE_{ij} kcal·mol^{-1}	NLMO %parent NBO// composition %
1	LP N (sp3) / 1.66	LV Ge (p) / 0.37	131.9	82.1 / 82.1 N, 14.4 Ge, 0.5 Ir_{98}
2	LP Cl (p) / 1.74	BD σ* 0.60 Ge (sp) – 0.8 Ir_{98} (sp) / 0.41	11.8	86.4 / 86.4 Cl, 4.1 Ir 98, 4.3 Ir 99, 0.3 P
3		BD σ* 0.75 C_{32} (sp3) – 0.66 Ir_{98} (sd3) / 0.36	30.4	
4		BD σ* 0.74 C_{36} (sp3) – 0.68 Ir_{98} (sd3) / 0.39	20.4	
5		BD σ* 0.57 P (sp2) – 0.82 Ir_{99} (sd) / 0.39	10.4	
6		BD σ* 0.74 C_{20} (sp3) – 0.67 Ir_{99} (sd3) / 0.38	17.0	
7		BD σ* 0.75 C_{39} (sp3) – 0.66 Ir_{99} (sd3) / 0.38	34.5	
8	LP Cl (sp2) / 1.62	BD σ* 0.60 Ge (sp) – 0.8 Ir_{98} (sp) / 0.41	6.1	80.5 / 80.5 Cl, 6.5 Ir_{98}, 6.4 Ir_{99}, 0.5 Ge
9		BD σ* 0.75 C_{32} (sp3) – 0.66 Ir_{98} (sd3) / 0.36	23.8	

10		BD σ* 0.74 C $_{36}$ (sp3) – 0.68 Ir $_{98}$ (sd3) / 0.39	46.8	
11		BD σ* 0.57 P (sp2) – 0.82 Ir $_{99}$ (sd) / 0.39	5.6	
12		BD σ* 0.74 C $_{20}$ (sp3) – 0.67 Ir $_{99}$ (sd3) / 0.38	44.6	
13		BD σ* 0.75 C $_{39}$ (sp3) – 0.66 Ir $_{99}$ (sd3) / 0.38	31.5	
14	BD σ 0.80 Ge (sp) + 0.60 Ir $_{98}$ (sd) / 1.86			
15	BD σ 0.45 Ge (sp3) + 0.89 Cl (sp3) / 1.97			
16	BD σ 0.47 Ge (sp3) + 0.88 Cl (sp3) / 1.97			
17	BD σ 0.57 Rh (sd) + 0.82 P (sp^2) / 1.88			

[a]LP = Lone Pair. [b]BD = bonding (2c-2e). [c]LV = Lone Vacancy.

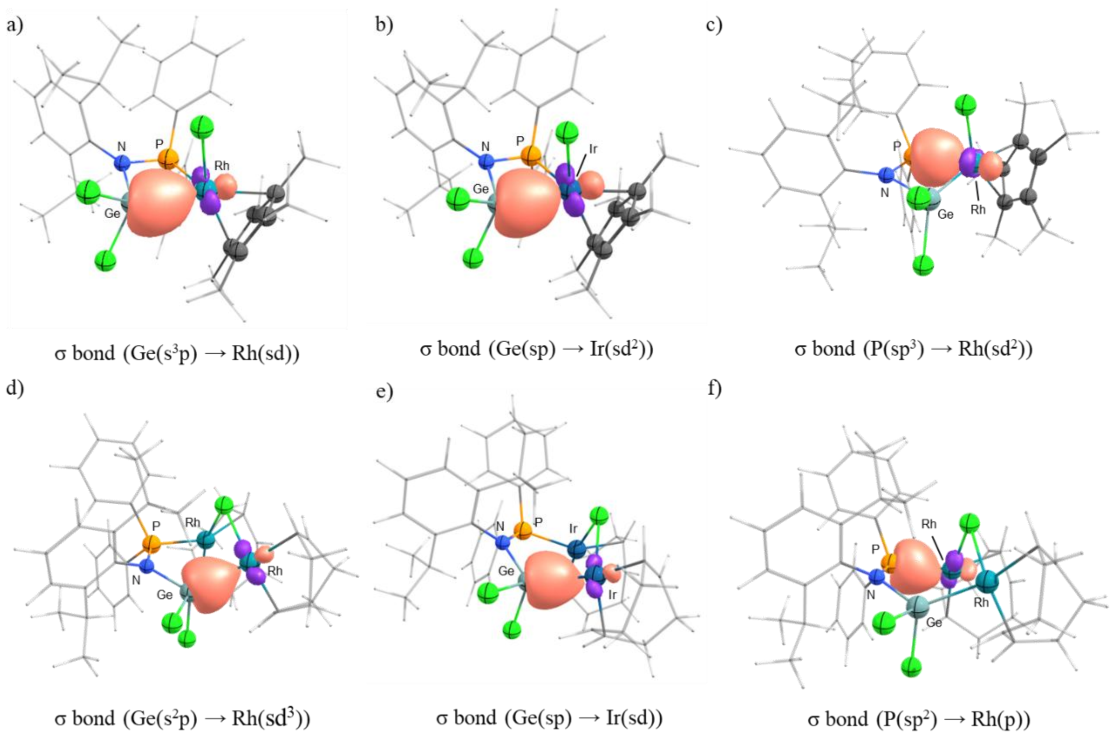

Figure S15. Selected NLMO representations, a) table S2 entry 5, b) table S3 entry 5, c) table S2 entry 6, d) table S4 entry 8, e) table S5 entry 14, and f) table S4 entry 10.

Table S6. Wiberg Bond Indices.

		**1a**	**1b**	**2a**	**2b**
**bond**	Rh/Ir—Ge	0.47	0.53	0.523	0.556
	Rh/Ir—P	0.53	0.60	0.5529	0.587
	Ge—N	0.53	0.54	0.499	0.51
	Ge—Cl	0.795	0.795	0.685	0.74
	Ge—Cl	0.71	0.716	0.729	0.70

The **frontier molecular orbitals** of complexes **1** were calculated to be qualitatively similar, particularly their HOMOs, which correspond primarily to an antibonding combination of a metal-centered *d* hybrid with one *p* orbital localized on the metal-bound chlorine atom (Figure S14). The LUMO of **1a** corresponds to an antibonding combination of a *d* hybrid localized on the transition metal ion and a *sp* hybrid on the germanium atom, whereas in **1b** the germanium atom has no significant contribution, replaced by orbitals of the P—Ph linkage.

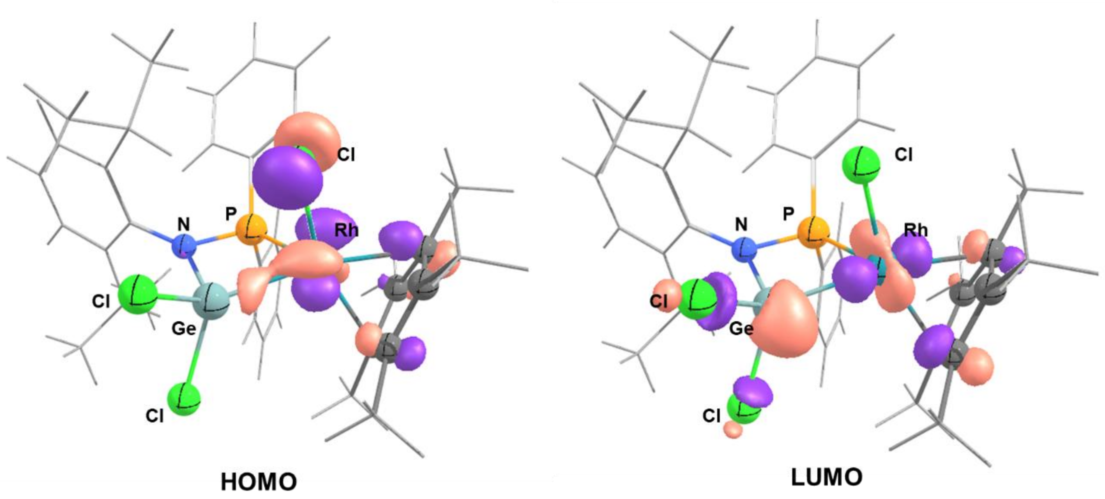

Figure S16. Frontier orbitals (0.07 a.u. isosurface) calculated for species **1a**.

In species **2** the HOMO is metal-centered, while the LUMO is related to π P—M and Ge—M interactions, with the former being more important in **2b**.

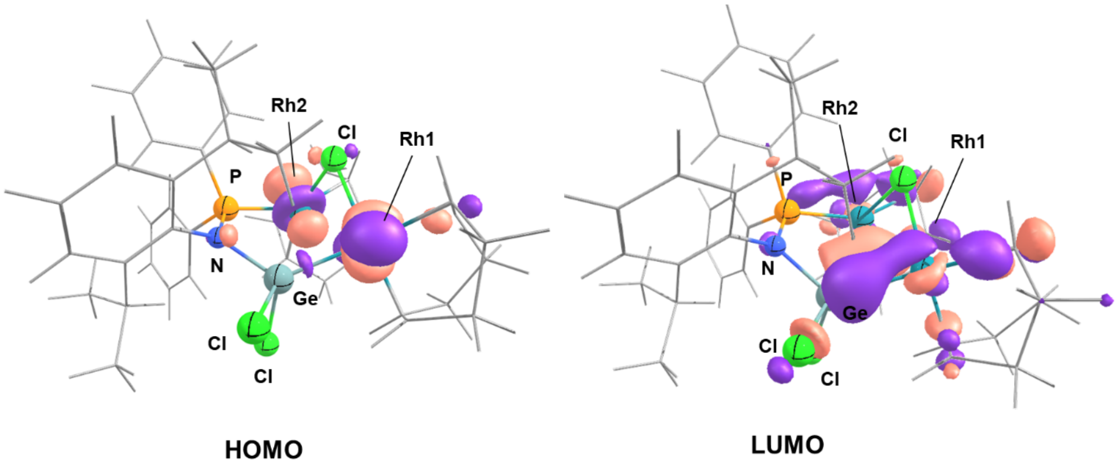

Figure S17. Frontier orbitals (HOMO 0.07 a.u. isosurface, LUMO 0.05 a.u. isosurface) calculated for species **2a**.

6. References

1. Stasch, A. A Hydrocarbon-Soluble Lithium Hydride Complex. *Angew. Chem. Int. Ed.* **2012**, *51*, 1930 –1933.
2. White, C.; Yates, A.; Maitlis, P. M.; Heinekey, D. M. (η^5-Pentamethylcyclopentadienyl)Rhodium and -Iridium Compounds. *Inorg. Synth.*, **1992**, *29*, 228-234.
3. Giordano, G.; Crabtree, R. H.; Heintz, R. M.; Forster, D.; Morris, D. E. Di-μ-Chloro-Bis(η^4-1,5-Cyclooctadiene)-Dirhodium(I). *Inorg. Synth.*, **1990**, *19*, 218–220.
4. Sheldrick, G. M. A Short History of SHELX. *Acta Cryst.* **2008**, A64, 112.
5. Gaussian 16, Revision C.01, M. J. Frisch, G. W. Trucks, H. B. Schlegel, G. E. Scuseria, M. A. Robb, J. R. Cheeseman, G. Scalmani, V. Barone, G. A. Petersson, H. Nakatsuji, X. Li, M. Caricato, A. V. Marenich, J. Bloino, B. G. Janesko, R. Gomperts, B. Mennucci, H. P. Hratchian, J. V. Ortiz, A. F. Izmaylov, J. L. Sonnenberg, D. Williams-Young, F. Ding, F. Lipparini, F. Egidi, J. Goings, B. Peng, A. Petrone, T. Henderson, D. Ranasinghe, V. G. Zakrzewski, J. Gao, N. Rega, G. Zheng, W. Liang, M. Hada, M. Ehara, K. Toyota, R. Fukuda, J. Hasegawa, M. Ishida, T. Nakajima, Y. Honda, O. Kitao, H. Nakai, T. Vreven, K. Throssell, J. A. Montgomery, Jr., J. E. Peralta, F. Ogliaro, M. J. Bearpark, J. J. Heyd, E. N. Brothers, K. N. Kudin, V. N. Staroverov, T. A. Keith, R. Kobayashi, J. Normand, K. Raghavachari, A. P. Rendell, J. C. Burant, S. S. Iyengar, J. Tomasi, M. Cossi, J. M. Millam, M. Klene, C. Adamo, R. Cammi, J. W. Ochterski, R. L. Martin, K. Morokuma, O. Farkas, J. B. Foresman, and D. J. Fox, Gaussian, Inc., Wallingford CT, 2016.
6. J. P. Perdew, in *Electronic Structure of Solids '91*, Ed. P. Ziesche and H. Eschrig (Akademie Verlag, Berlin, **1991**, 11.
7. S. Grimme, S. Ehrlich and L. Goerigk, *J. Comp. Chem.* 2011, **32**, 1456-65. DOI: 10.1002/jcc.21759
8. a) R. Ditchfield, W. J. Hehre, J. A. Pople, *J. Chem. Phys.* 1971, **54**, 724–728; b) W. J. Hehre, R. Ditchfield, J. A. Pople, *J. Chem. Phys.* 1972, **56**, 2257–2261; c) P. C. Hariharan, J. A. Pople, *Theor. Chim. Acta* 1973, **28**, 213–222; d) M. M. Francl, W. J. Pietro, W. J. Hehre, J. S. Binkley, M. S. Gordon, D. J. DeFrees, J. A. Pople, *J. Chem. Phys.* 1982, **77**, 3654–3665; e) F. Weigend, R. Ahlrichs, *Phys. Chem. Chem. Phys.*, **2005**, *7*, 3297-305. DOI: 10.1039/B508541A

9. D. Andrae, U. Haeussermann, M. Dolg, H. Stoll, H. Preuss, *Theor. Chem. Acc.*, **1990**, ***77,*** 123-41. DOI: 10.1007/BF01114537
10. A. V. Marenich, C. J. Cramer, D. G. Truhlar, *J. Phys. Chem. B* 2009, **113**, 6378–6396.
11. D. Andrae, U. Häußermann, M. Dolg, H. Stoll, H. Preuß, *Theor. Chim. Acta* **1990**, *77*, 123-141. DOI: 10.1007/BF01114537
12. a) E. D. Glendening, C. R. Landis, F. Weinhold, *J. Comput. Chem.* 2013, **34**, 1429-1437; b) E. D. Glendening, J. K. Badenhoop, A. E. Reed, J. E. Carpenter, J. A. Bohmann, C. M. Morales, C. R. Landis, F. Weinhold, "NBO 6.0." Theoretical Chemistry Institute, University of Wisconsin: Madison, 2013. Available at: https://chem.wisc.edu/
